# Supplementary material for: A Critical View of the Application of the APEX Software (Aqueous Photochemistry of Environmentally-Occurring Xenobiotics) to Predict Photoreaction Kinetics in Surface Freshwaters
Source: Molecules. 2019 Dec 18;25(1):9. doi: 10.3390/molecules25010009 (PMC7017383; doi:10.3390/molecules25010009)
Supplement: Supplementary file 1 [file molecules-25-00009-s001.zip › 3.2.4_gcc-4.4.0.zip!/3.2.4_gcc-4.4.0/3.2.4_gcc-4.4.0/share/octave/packages/communications-1.0.10/doc/comms.pdf]

Communications Toolbox for Octave

March 2003

David Bateman
Laurent Mazet
Paul Kienzle

Copyright © 2003

Permission is granted to make and distribute verbatim copies of this manual provided the copyright notice and this permission notice are preserved on all copies.

Permission is granted to copy and distribute modified versions of this manual under the conditions for verbatim copying, provided that the entire resulting derived work is distributed under the terms of a permission notice identical to this one.

Permission is granted to copy and distribute translations of this manual into another language, under the same conditions as for modified versions.

Table of Contents

1 Introduction 1

2 Random Signals 2

2.1 Signal Creation 2
2.2 Signal Analysis 5

3 Source Coding 9

3.1 Quantization 9
3.2 PCM Coding 9
3.3 Arithmetic Coding 10
3.4 Dynamic Range Compression 10

4 Block Coding 11

4.1 Data Formats 11
4.2 Binary Block Codes 12
4.3 BCH Codes 14
4.4 Reed-Solomon Codes 16
4.4.1 Representation of Reed-Solomon Messages 16
4.4.2 Creating and Decoding Messages 17
4.4.3 Shortened Reed-Solomon Codes 18

5 Convolutional Coding 20

6 Modulations 21

7 Special Filters 22

8 Galois Fields 23

8.1 Galois Field Basics 23
8.1.1 Creating Galois Fields 23
8.1.2 Primitive Polynomials 24
8.1.3 Accessing Internal Fields 26
8.1.4 Function Overloading 26
8.1.5 Known Problems 27
8.2 Manipulating Galois Fields 28
8.2.1 Expressions, manipulation and assignment 28
8.2.2 Unary operations 30
8.2.3 Arithmetic operations 31
8.2.4 Comparison operations 32
8.2.5 Polynomial manipulations 32
8.2.6 Linear Algebra 36
8.2.7 Signal Processing with Galois Fields 36

9 Function Reference . . . 38
9.1 Functions by Category . . . 38
9.1.1 Random Signals . . . 38
9.1.2 Source Coding . . . 38
9.1.3 Block Interleavers . . . 40
9.1.4 Block Coding . . . 40
9.1.5 Modulations . . . 41
9.1.6 Special Filters . . . 42
9.1.7 Galois Fields of Even Characateristic . . . 43
9.1.8 Galois Fields of Odd Characteristic . . . 44
9.1.9 Utility Functions . . . 45
9.2 Functions Alphabetically . . . 45
9.2.1 ademodce . . . 45
9.2.2 amdemod . . . 46
9.2.3 ammod . . . 46
9.2.4 amodce . . . 47
9.2.5 apkconst . . . 47
9.2.6 awgn . . . 48
9.2.7 bchdeco . . . 49
9.2.8 bchenco . . . 49
9.2.9 bchpoly . . . 50
9.2.10 bi2de . . . 51
9.2.11 biterr . . . 51
9.2.12 bsc . . . 52
9.2.13 comms . . . 52
9.2.14 compand . . . 53
9.2.15 cosets . . . 54
9.2.16 cyclgen . . . 54
9.2.17 cyclpoly . . . 54
9.2.18 de2bi . . . 55
9.2.19 decode . . . 55
9.2.20 deintrlv . . . 56
9.2.21 demodmap . . . 57
9.2.22 egolaydec . . . 58
9.2.23 egolayenc . . . 58
9.2.24 egolaygen . . . 58
9.2.25 encode . . . 59
9.2.26 eyediagram . . . 60
9.2.27 fibodeco . . . 60
9.2.28 fiboenco . . . 61
9.2.29 fibosplitstream . . . 61
9.2.30 fmdemod . . . 62
9.2.31 fmmod . . . 62
9.2.32 gconv . . . 62
9.2.33 gconvmtx . . . 62
9.2.34 gdeconv . . . 63
9.2.35 gdet . . . 63
9.2.36 gdftmtx . . . 63

9.2.37 gdiag . . . 63
9.2.38 gen2par . . . 64
9.2.39 genqamdemod . . . 64
9.2.40 genqammod . . . 64
9.2.41 gexp . . . 64
9.2.42 gf . . . 65
9.2.43 gfft . . . 65
9.2.44 gfilter . . . 65
9.2.45 gftable . . . 66
9.2.46 gfweight . . . 66
9.2.47 gifft . . . 66
9.2.48 ginv . . . 66
9.2.49 ginverse . . . 66
9.2.50 gisequal . . . 67
9.2.51 glog . . . 67
9.2.52 glu . . . 67
9.2.53 golombdeco . . . 68
9.2.54 golombenco . . . 68
9.2.55 gprod . . . 69
9.2.56 grank . . . 69
9.2.57 greshape . . . 69
9.2.58 groots . . . 69
9.2.59 gsqrt . . . 70
9.2.60 gsum . . . 70
9.2.61 gsumsq . . . 70
9.2.62 hammgen . . . 70
9.2.63 huffmandeco . . . 71
9.2.64 huffmandict . . . 71
9.2.65 huffmanenco . . . 72
9.2.66 intrlv . . . 72
9.2.67 isgalois . . . 72
9.2.68 isprimitive . . . 72
9.2.69 lloyds . . . 73
9.2.70 lz77deco . . . 73
9.2.71 lz77enco . . . 74
9.2.72 marcumq . . . 74
9.2.73 matdeintrlv . . . 74
9.2.74 matintrlv . . . 74
9.2.75 minpol . . . 74
9.2.76 modmap . . . 74
9.2.77 oct2dec . . . 76
9.2.78 pamdemod . . . 76
9.2.79 pammod . . . 76
9.2.80 primpoly . . . 76
9.2.81 pskdemod . . . 77
9.2.82 pskmod . . . 77
9.2.83 qamdemod . . . 78
9.2.84 qammod . . . 78

9.2.85 qaskdeco 78
9.2.86 qaskenco 79
9.2.87 qfunc 79
9.2.88 qfuncinv 79
9.2.89 quantiz 80
9.2.90 randdeintrlv 80
9.2.91 randerr 80
9.2.92 randint 81
9.2.93 randintrlv 81
9.2.94 randsrc 81
9.2.95 reedmullerdec 81
9.2.96 reedmullerenc 82
9.2.97 reedmullergen 82
9.2.98 ricedeco 83
9.2.99 riceenco 83
9.2.100 rledeco 83
9.2.101 rleenco 84
9.2.102 rsdec 84
9.2.103 rsdecof 85
9.2.104 rsenc 85
9.2.105 rsencof 86
9.2.106 rsgenpoly 86
9.2.107 scatterplot 87
9.2.108 shannonfanodeco 87
9.2.109 shannonfanodict 88
9.2.110 shannonfanoenco 88
9.2.111 symerr 88
9.2.112 syndtable 89
9.2.113 systematize 89
9.2.114 vec2mat 89
9.2.115 wgn 90

1 Introduction

This is the start of documentation for a Communications Toolbox for Octave. As functions are written they should be documented here. In addition many of the existing functions of Octave and Octave-Forge are important in this Toolbox and their documentation should perhaps be repeated here.

This is preliminary documentation and you are invited to improve it and submit patches.

2 Random Signals

The purpose of the functions described here is to create and add random noise to a signal, to create random data and to analyze the eventually errors in a received signal. The functions to perform these tasks can be considered as either related to the creation or analysis of signals and are treated separately below.

It should be noted that the examples below are based on the output of a random number generator, and so the user can not expect to exactly recreate the examples below.

2.1 Signal Creation

The signal creation functions here fall into to two classes. Those that treat discrete data and those that treat continuous data. The basic function to create discrete data is *randint*, that creates a random matrix of equi-probable integers in a desired range. For example

```
octave:1> a = randint(3,3,[-1,1])
a =

   0   1   0
  -1  -1   1
   0   1   1
```

creates a 3-by-3 matrix of random integers in the range -1 to 1. To allow for repeated analysis with the same random data, the function *randint* allows the seed-value of the random number generator to be set. For instance

```
octave:1> a = randint(3,3,[-1,1],1)
a =

   0   1   1
   0  -1   0
   1  -1  -1
```

will always produce the same set of random data. The range of the integers to produce can either be a two element vector or an integer. In the case of a two element vector all elements within the defined range can be produced. In the case of an integer range *M*, *randint* returns the equi-probable integers in the range $[0 : 2^m - 1]$.

The function *randsrc* differs from *randint* in that it allows a random set of symbols to be created with a given probability. The symbols can be real, complex or even characters. However characters and scalars can not be mixed. For example

```
octave:1> a = randsrc(2,2,"ab");
octave:2> b = randsrc(4,4,[1, 1i, -1, -1i,]);
```

are both legal, while

```
octave:1> a = randsrc(2,2,[1,"a"]);
```

is not legal. The alphabet from which the symbols are chosen can be either a row vector or two row matrix. In the case of a row vector, all of the elements of the alphabet are chosen with an equi-probability. In the case of a two row matrix, the values in the second row define the probability that each of the symbols are chosen. For example

```
octave:1> a = randsrc(5,5,[1, 1i, -1, -1i; 0.6 0.2 0.1 0.1])
a =

   1 + 0i   0 + 1i   0 + 1i   0 + 1i   1 + 0i
   1 + 0i   1 + 0i   0 + 1i   0 + 1i   1 + 0i
  -0 - 1i   1 + 0i  -1 + 0i   1 + 0i   0 + 1i
   1 + 0i   1 + 0i   1 + 0i   1 + 0i   1 + 0i
  -1 + 0i  -1 + 0i   1 + 0i   1 + 0i   1 + 0i
```

defines that the symbol '1' has a 60% probability, the symbol '1i' has a 20% probability and the remaining symbols have 10% probability each. The sum of the probabilities must equal one. Like *randint*, *randsrc* accepts a fourth argument as the seed of the random number generator allowing the same random set of data to be reproduced.

The function *randerr* allows a matrix of random bit errors to be created, for binary encoded messages. By default, *randerr* creates exactly one errors per row, flagged by a non-zero value in the returned matrix. That is

```
octave:1> a = randerr(5,10)
a =

  0  1  0  0  0  0  0  0  0  0
  0  0  1  0  0  0  0  0  0  0
  0  0  1  0  0  0  0  0  0  0
  0  0  0  0  0  0  0  0  0  1
  0  0  0  0  0  0  0  0  0  1
```

The number of errors per row can be specified as the third argument to *randerr*. This argument can be either a scalar, a row vector or a two row matrix. In the case of a scalar value, exactly this number of errors will be created per row in the returned matrix. In the case of a row vector, each element of the row vector gives a possible number of equi-probable bit errors. The second row of a two row matrix defines the probability of each number of errors occurring. For example

```
octave:1> n = 15; k = 11; nsym = 100;
octave:2> msg = randint(nsym,k);       ## Binary vector of message
octave:3> code = encode(msg,n,k,"bch");
octave:4> berrs = randerr(nsym,n,[0, 1; 0.7, 0.3]);
octave:5> noisy = mod(code + berrs, 2) ## Add errors to coded message
```

creates a vector *msg*, encodes it with a [15,11] BCH code, and then add either none or one error per symbol with the chances of an error being 30%. As previously, *randerr* accepts a fourth argument as the seed of the random number generator allowing the same random set of data to be reproduced.

All of the above functions work on discrete random signals. The functions *wgn* and *awgn* create and add white Gaussian noise to continuous signals. The function *wgn* creates a matrix of white Gaussian noise of a certain power. A typical call to *wgn* is then

```
octave:1> nse = wgn(10,10,0);
```

Which creates a 10-by-10 matrix of noise with a root mean squared power of 0dBW relative to an impedance of 1Ω.

This effectively means that an equivalent result to the above can be obtained with

```
octave:1> nse = randn(10,10);
```

The reference impedance and units of power to the function *wgn* can however be modified, for example

```
octave:1> nse_30dBm_50Ohm = wgn(10000,1,30,50,"dBm");
octave:2> nse_0dBW_50Ohm = wgn(10000,1,0,50,"dBW");
octave:3> nse_1W_50Ohm = wgn(10000,1,1,50,"linear");
octave:4> [std(nse_30dBm_50Ohm), std(nse_0dBW_50Ohm), std(nse_1W_50Ohm)]
ans =

  7.0805  7.1061  7.0730
```

All produce a 1W signal referenced to a 50Ω. impedance. Matlab uses the misnomer "dB" for "dBW", and therefore "dB" is an accepted type for *wgn* and will be treated as for "dBW".

In all cases, the returned matrix *v*, will be related to the input power *p* and the impedance *Z* as

$$p = \frac{\sum_i \sum_j v(i,j)^2}{Z} Watts$$

By default *wgn* produces real vectors of white noise. However, it can produce both real and complex vectors like

```
octave:1> rnse = wgn(10000,1,0,"dBm","real");
octave:2> cnse = wgn(10000,1,0,"dBm","complex");
octave:3> [std(rnse), std(real(cnse)), std(imag(cnse)), std(cnse)]
ans =

  0.031615  0.022042  0.022241  0.031313
```

which shows that with a complex return value that the total power is the same as a real vector, but that it is equally shared between the real and imaginary parts. As previously, *wgn* accepts a fourth numerical argument as the seed of the random number generator allowing the same random set of data to be reproduced. That is

```
octave:1> nse = wgn(10,10,0,0);
```

will always produce the same set of data.

The final function to deal with the creation of random signals is *awgn*, that adds noise at a certain level relative to a desired signal. This function adds noise at a certain level to a desired signal. An example call to *awgn* is

```
octave:1> x = [0:0.1:2*pi];
octave:2> y = sin(x);
octave:3> noisy = awgn(y, 10, "measured")
```

which produces a sine-wave with noise added as seen in Figure 1.

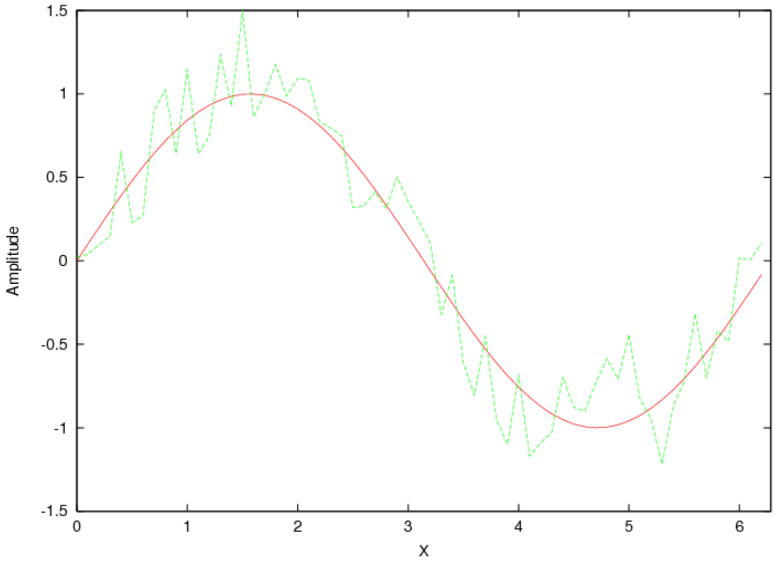

Figure 1: Sine-wave with 10dB signal-to-noise ratio

which adds noise with a 10dB signal-to-noise ratio to the measured power in the desired signal. By default *awgn* assumes that the desired signal is at 0dBW, and the noise is added relative to this assumed power. This behavior can be modified by the third argument to *awgn*. If the third argument is a numerical value, it is assumed to define the power in the input signal, otherwise if the third argument is the string 'measured', as above, the power in the signal is measured prior to the addition of the noise.

The final argument to *awgn* defines the definition of the power and signal-to-noise ratio in a similar manner to *wgn*. This final argument can be either 'dB' or 'linear'. In the first case the numerical value of the input power is assumed to be in dBW and the signal-to-noise ratio in dB. In the second case, the power is assumed to be in Watts and the signal-to-noise ratio is expressed as a ratio.

The return value of *awgn* will be in the same form as the input signal. In addition if the input signal is real, the additive noise will be real. Otherwise the additive noise will also be complex and the noise will be equally split between the real and imaginary parts.

As previously the seed to the random number generator can be specified as the last argument to *awgn* to allow repetition of the same scenario. That is

```
octave:1> x = [0:0.1:2*pi];
octave:2> y = sin(x);
octave:3> noisy = awgn(y, 10, "dB", 0, "measured")
```

which uses the seed-value of 0 for the random number generator.

2.2 Signal Analysis

It is important to be able to evaluate the performance of a communications system in terms of its bit-error and symbol-error rates. Two functions *biterr* and *symerr* exist within this package to calculate these values, both taking as arguments the expected and the actually received data. The data takes the form of matrices or vectors, with each element representing a single symbol. They are compared in the following manner

Both matrices
: In this case both matrices must be the same size and then by default the the return values are the overall number of errors and the overall error rate.

One column vector
: In this case the column vector is used for comparison column-wise with the matrix. The return values are row vectors containing the number of errors and the error rate for each column-wise comparison. The number of rows in the matrix must be the same as the length of the column vector.

One row vector
: In this case the row vector is used for comparison row-wise with the matrix. The return values are column vectors containing the number of errors and the error rate for each row-wise comparison. The number of columns in the matrix must be the same as the length of the row vector.

For the bit-error comparison, the size of the symbol is assumed to be the minimum number of bits needed to represent the largest element in the two matrices supplied. However, the number of bits per symbol can (and in the case of random data should) be specified. As an example of the use of *biterr* and *symerr*, consider the example

```
octave:1> m = 8;
octave:2> msg = randint(10,10,2^m);
octave:3> noisy = mod(msg + diag(1:10),2^m);
octave:4> [berr, brate] = biterr(msg, noisy, m)
berr = 32
brate = 0.040000
octave:5> [serr, srate] = symerr(msg, noisy)
serr = 10
srate = 0.10000
```

which creates a 10-by-10 matrix adds 10 symbols errors to the data and then finds the bit and symbol error-rates.

Two other means of displaying the integrity of a signal are the eye-diagram and the scatterplot. Although the functions *eyediagram* and *scatterplot* have different appearance, the information presented is similar and so are their inputs. The difference between *eyediagram* and *scatterplot* is that *eyediagram* segments the data into time intervals and plots the in-phase and quadrature components of the signal against this time interval. While *scatterplot* uses a parametric plot of quadrature versus in-phase components.

Both functions can accept real or complex signals in the following forms.

A real vector
: In this case the signal is assumed to be real and represented by the vector *x*.

A complex vector
: In this case the in-phase and quadrature components of the signal are assumed to be the real and imaginary parts of the signal.

A matrix with two columns
: In this case the first column represents the in-phase and the second the quadrature components of a complex signal.

An example of the use of the function *eyediagram* is

```
octave:1> n = 50;
octave:2> ovsp=50;
octave:3> x = 1:n;
octave:4> xi = [1:1/ovsp:n-0.1];
octave:5> y = randsrc(1,n,[1 + 1i, 1 - 1i, -1 - 1i, -1 + 1i]) ;
octave:6> yi = interp1(x,y,xi);
octave:7> noisy = awgn(yi,15,"measured");
octave:8> eyediagram(noisy,ovsp);
```

which produces a eye-diagram of a noisy signal as seen in Figure 2. Similarly an example of the use of the function *scatterplot* is

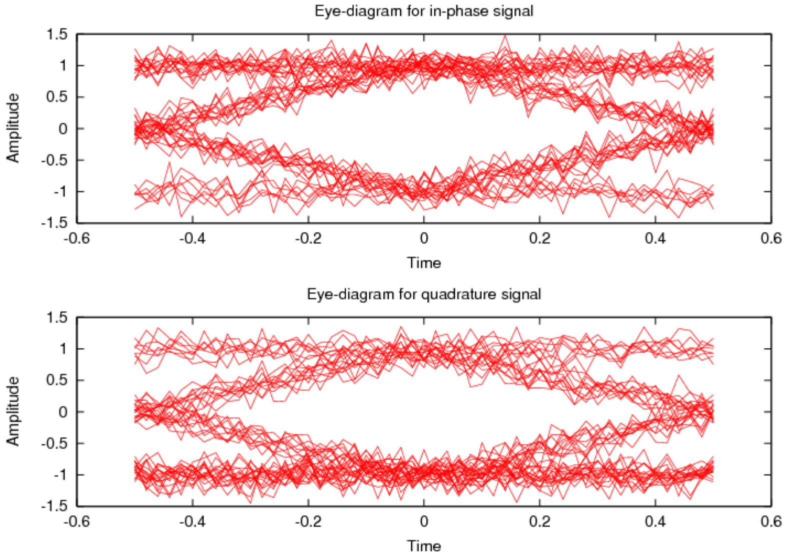

Figure 2: Eye-diagram of a QPSK like signal with 15dB signal-to-noise ratio

```
octave:1> n = 200;
octave:2> ovsp=5;
octave:3> x = 1:n;
octave:4> xi = [1:1/ovsp:n-0.1];
octave:5> y = randsrc(1,n,[1 + 1i, 1 - 1i, -1 - 1i, -1 + 1i]) ;
octave:6> yi = interp1(x,y,xi);
octave:7> noisy = awgn(yi,15,"measured");
octave:8> hold off;
octave:9> scatterplot(noisy,1,0,"b");
octave:10> hold on;
octave:11> scatterplot(noisy,ovsp,0,"r+");
```

which produces a scatterplot of a noisy signal as seen in Figure 3.

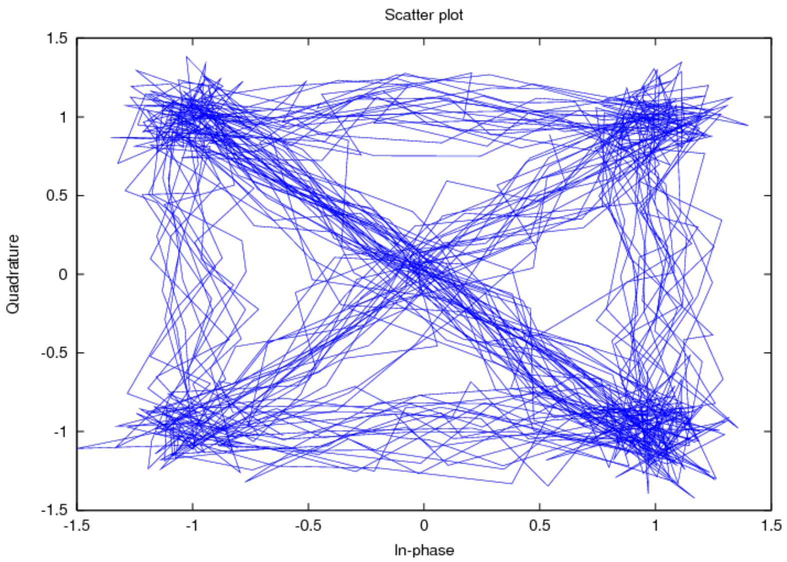

Figure 3: Scatterplot of a QPSK like signal with 15dB signal-to-noise ratio

3 Source Coding

3.1 Quantization

An important aspect of converting an analog signal to the digital domain is quantization. This is the process of mapping a continuous signal to a set of defined values. Octave contains two functions to perform quantization, *lloyds* creates an optimal mapping of the continous signal to a fixed number of levels and *quantiz* performs the actual quantization.

The set of quantization points to use is represented by a partitioning table (*table*) of the data and the signal levels (*codes* to which they are mapped. The partitioning *table* is monotonicly increasing and if x falls within the range given by two points of this table then it is mapped to the corresponding code as seen in Table 1.

Table 1: Table quantization partitioning and coding

x < table(1)	codes(1)
table(1) <= x < table(2)	codes(2)
...	...
table(i-1) <= x < table(i)	codes(i)
...	...
table(n-1) <= x < table(n)	codes(n)
table(n-1) <= x	codes(n+1)

These partition and coding tables can either be created by the user of using the function *lloyds*. For instance the use of a linear mapping can be seen in the following example.

```
octave:1> m = 8;
octave:2> n = 1024;
octave:3> table = 2*[0:m-1]/m - 1 + 1/m;
octave:4> codes = 2*[0:m]/m - 1;
octave:5> x = 4*pi*[0:(n-1)]/(n-1);
octave:6> y = cos(x);
octave:7> [i,z] = quantiz(y, table, codes);
```

If a training signal is known that well represents the expected signals, the quantization levels can be optimized using the *lloyds* function. For example the above example can be continued

```
octave:8> [table2, codes2] = lloyds(y, table, codes);
octave:9> [i,z2] = quantiz(y, table2, codes2);
```

Which the mapping suggested by the function *lloyds*. It should be noted that the mapping given by *lloyds* is highly dependent on the training signal used. So if this signal does not represent a realistic signal to be quantized, then the parititioning suggested by *lloyds* will be sub-optimal.

3.2 PCM Coding

The DPCM function *dpcmenco*, *dpcmdeco* and *dpcmopt* implement a form of preditive quantization, where the predictability of the signal is used to further compress it. These functions are not yet implemented.

3.3 Arithmetic Coding

The arithmetic coding functions *arithenco* and *arithdeco* are not yet implemented.

3.4 Dynamic Range Compression

The final source coding function is *compand* which is used to compress and expand the dynamic range of a signal. For instance consider a logarithm quantized by a linear partitioning. Such a partitioning is very poor for this large dynamic range. *compand* can then be used to compress the signal prior to quantization, with the signal being expanded afterwards. For example

```
octave:1> mu = 1.95;
octave:2> x = [0.01:0.01:2];
octave:3> y = log(x);
octave:4> V = max(abs(y));
octave:5> [i,z,d] = quantiz(y,[-4.875:0.25:0.875],[-5:0.25:1]);
octave:6> c = compand(y,minmu,V,'mu/compressor');
octave:7> [i2,c2] = quantiz(c,[-4.875:0.25:0.875],[-5:0.25:1]);
octave:8> z2 = compand(c2,minmu,max(abs(c2)),'mu/expander');
octave:9> d2 = sumsq(y-z2) / length(y);
octave:10> [d, d2]
ans =

  0.0053885  0.0029935
```

which demonstrates that the use of *compand* can significantly reduce the distortion due to the quantization of signals with a large dynamic range.

4 Block Coding

The error-correcting codes available in this toolbox are discussed here. These codes work with blocks of data, with no relation between one block and the next. These codes create codewords based on the messages to transmit that contain redundant information that allow the recovery of the original message in the presence of errors.

4.1 Data Formats

All of the codes described in this section are binary and share similar data formats. The exception is the Reed-Solomon coder which has a significantly longer codeword length in general and therefore using a different manner to efficiently pass data. The user should reference to the section about the Reed-Solomon codes for the data format for use with Reed-Solomon codes.

In general k bits of data are considered to represent a single message symbol. These k bits are coded into n bits of data representing the codeword. The data can therefore be grouped in one of three manners, to emphasis this grouping into bits, messages and codewords

A binary vector
: Each element of the vector is either one or zero. If the data represents an uncoded message the vector length should be an integer number of k in length.

A binary matrix
: In this case the data is ones and zeros grouped into rows, with each representing a single message or codeword. The number of columns in the matrix should be equal to k in the case of a uncoded message or n in the case of a coded message.

A non-binary vector
: In this case each element of the vector represents a message or codeword in an integer format. The bits of the message or codeword are represented by the bits of the vector elements with the least-significant bit representing the first element in the message or codeword.

An example demonstrating the relationship between the three data formats can be seen below.

```
octave:1> k = 4;
octave:2> bin_vec = randint(k*10,1);         # Binary vector format
octave:3> bin_mat = reshape(bin_vec,k,10)'; # Binary matrix format
octave:4> dec_vec = bi2de(bin_mat);          # Decimal vector format
```

The functions within this toolbox will return data in the same format to which it is given. It should be noted that internally the binary matrix format is used, and thus if the message or codeword length is large it is preferable to use the binary format to avoid internal rounding errors.

4.2 Binary Block Codes

All of the codes presented here can be characterized by their

Generator Matrix
: A k-by-n matrix G to generate the codewords C from the messages T by the matrix multiplication $\mathbf{C} = \mathbf{TG}$.

Parity Check Matrix
: A 'n-k'-by-n matrix H to check the parity of the received symbols. If $\mathbf{HR} = \mathbf{S} \neq 0$, then an error has been detected. S can be used with the syndrome table to correct this error

Syndrome Table
: A 2^k-by-n matrix ST with the relationship of the error vectors to the non-zero parities of the received symbols. That is, if the received symbol is represented as $\mathbf{R} = (\mathbf{T} + \mathbf{E})$ *mod* 2, then the error vector E is $\mathbf{ST}(\mathbf{S})$.

It is assumed for most of the functions in this toolbox that the generator matrix will be in a 'standard' form. That is the generator matrix can be represented by

$$\mathbf{G} = \begin{bmatrix} g_{11} & g_{12} & \dots & g_{1k} & 1 & 0 & \dots & 0 \\ g_{21} & g_{22} & & g_{2k} & 0 & 1 & & 0 \\ \vdots & & & \vdots & \vdots & & & \vdots \\ g_{k1} & g_{k2} & \dots & g_{kk} & 0 & 0 & \dots & 1 \end{bmatrix}$$

or

$$\mathbf{G} = \begin{bmatrix} 1 & 0 & \dots & 0 & g_{11} & g_{12} & \dots & g_{1k} \\ 0 & 1 & & 0 & g_{21} & g_{22} & & g_{2k} \\ \vdots & & & \vdots & \vdots & & & \vdots \\ 0 & 0 & \dots & 1 & g_{k1} & g_{k2} & \dots & g_{kk} \end{bmatrix}$$

and similarly the parity check matrix can be represented by a combination of an identity matrix and a square matrix.

Some of the codes can also have their representation in terms of a generator polynomial that can be used to create the generator and parity check matrices. In the case of BCH codes, this generator polynomial is used directly in the encoding and decoding without ever explicitly forming the generator or parity check matrix.

The user can create their own generator and parity check matrices, or they can rely on the functions *hammgen*, *cyclgen* and *cyclpoly*. The function *hammgen* creates parity check and generator matrices for Hamming codes, while *cyclpoly* and *cyclgen* create generator polynomials and matrices for generic cyclic codes. An example of their use is

```
octave:1> m = 3;
octave:2> n = 2^m -1;
octave:2> k = 4;
octave:3> [par, gen] = hammgen(m);
octave:4> [par2, gen2] = cyclgen(n,cyclpoly(n,k));
```

which create identical parity check and generator matrices for the [7,4] Hamming code.

The syndrome table of the codes can be created with the function *syndtable*, in the following manner

```
octave:1> [par, gen] = hammgen(3);
octave:2> st = syndtable(par);
```

There exists two auxiliary functions *gen2par* and *gfweight*, that convert between generator and parity check matrices and calculate the Hamming distance of the codes. For instance

```
octave:1> par = hammgen(3);
octave:2> gen = gen2par(par);
octave:3> gfweight(gen)
ans = 3
```

It should be noted that for large values of *n*, the generator, parity check and syndrome table matrices are very large. There is therefore an internal limitation on the size of the block codes that can be created that limits the codeword length *n* to less than 64. Which is still excessively large for the syndrome table, so use caution with these codes. These limitations do not apply to the Reed-Solomon or BCH codes.

The top-level encode and decode functions are *encode* and *decode*, which can be used with all codes, except the Reed-Solomon code. The basic call to both of these functions passes the message to code/decode, the codeword length, the message length and the type of coding to use. There are four basic types that are available with these functions

'linear' Generic linear block codes

'cyclic' Cyclic linear block codes

'hamming' Hamming codes

'bch' Bose Chaudhuri Hocquenghem (BCH) block codes

It is not possible to distinguish between a binary vector and a decimal vector coding of the messages that just happens to only have ones and zeros. Therefore the functions *encode* and *decode* must be told the format of the messages in the following manner.

```
octave:1> m = 3;
octave:2> n = 7;
ocatve:3> k = 4;
octave:4> msg_bin = randint(10,k);
octave:5> cbin = encode(msg_bin, n, k, "hamming/binary");
octave:5> cdec = encode(bi2de(msg), n, k, "hamming/decimal");
```

which codes a binary matrix and a non-binary vector representation of a message, returning the coded message in the same format. The functions *encode* and *decode* by default accept binary coded messages. Therefore 'hamming' is equivalent to 'hamming/binary'.

Except for the BCH codes, the function *encode* and *decode* internally create the generator, parity check and syndrome table matrices. Therefore if repeated calls to *encode* and *decode* are made it will often be faster to create these matrices externally, and pass them as an argument. For example

```
n = 15;
k = 11;
[par, gen] = hammgen(4);
```

```
code1 = code2 = zeros(100,15)
for i=1:100
  msg = get_msg(i);
  code1(i,:) = encode(msg, n, k, 'linear', gen);  # This is faster
  code2(i,:) = encode(msg, n, k, 'hamming');      # than this !!!
end
```

In the case of the BCH codes the low-level functions described in the next section are used directly by the *encode* and *decode* functions.

4.3 BCH Codes

The BCH coder used here is based on code written by Robert Morelos-Zaragoza (r.morelos-zaragoza@ieee.org). This code was originally written in C and has been converted for use as an octave oct-file.

Called without arguments, *bchpoly* returns a table of valid BCH error correcting codes and their error-correction capability as seen in Table 1.

Table 2: Table of valid BCH codes with codeword length less than 511.

N	K	T	N	K	T	N	K	T	N	K	T
7	4	1	127	36	15	255	45	43	511	268	29
15	11	1	127	29	21	255	37	45	511	259	30
15	7	2	127	22	23	255	29	47	511	250	31
15	5	3	127	15	27	255	21	55	511	241	36
31	26	1	127	8	31	255	13	59	511	238	37
31	21	2	255	247	1	255	9	63	511	229	38
31	16	3	255	239	2	511	502	1	511	220	39
31	11	5	255	231	3	511	493	2	511	211	41
31	6	7	255	223	4	511	484	3	511	202	42
63	57	1	255	215	5	511	475	4	511	193	43
63	51	2	255	207	6	511	466	5	511	184	45
63	45	3	255	199	7	511	457	6	511	175	46
63	39	4	255	191	8	511	448	7	511	166	47
63	36	5	255	187	9	511	439	8	511	157	51
63	30	6	255	179	10	511	430	9	511	148	53
63	24	7	255	171	11	511	421	10	511	139	54
63	18	10	255	163	12	511	412	11	511	130	55
63	16	11	255	155	13	511	403	12	511	121	58
63	10	13	255	147	14	511	394	13	511	112	59
63	7	15	255	139	15	511	385	14	511	103	61
127	120	1	255	131	18	511	376	15	511	94	62
127	113	2	255	123	19	511	367	17	511	85	63
127	106	3	255	115	21	511	358	18	511	76	85
127	99	4	255	107	22	511	349	19	511	67	87
127	92	5	255	99	23	511	340	20	511	58	91
127	85	6	255	91	25	511	331	21	511	49	93
127	78	7	255	87	26	511	322	22	511	40	95
127	71	9	255	79	27	511	313	23	511	31	109

127	64	10	255	71	29	511	304	25	511	28	111
127	57	11	255	63	30	511	295	26	511	19	119
127	50	13	255	55	31	511	286	27	511	10	127
127	43	14	255	47	42	511	277	28			

The first returned column of *bchpoly* is the codeword length, the second the message length and the third the error correction capability of the code. Called with one argument, *bchpoly* returns similar output, but only for the specified codeword length. In this manner codes with codeword length greater than 511 can be found.

In general the codeword length is of the form `2^`*m*`-1`, where *m* is an integer. However if [*n*,*k*] is a valid BCH code, then it is also possible to use a shortened BCH form of the form `[`*n*`-`*x*`,`*k*`-`*x*`]`.

With two or more arguments, *bchpoly* is used to find the generator polynomial of a valid BCH code. For instance

```
octave:1> bchpoly(15,7)
ans =

  1  0  0  0  1  0  1  1  1

octave:2> bchpoly(14,6)
ans =

  1  0  0  0  1  0  1  1  1
```

show that the generator polynomial of a [15,7] BCH code with the default primitive polynomial is

$$1 + x^4 + x^6 + x^7 + x^8$$

Using a different primitive polynomial to define the Galois Field over which the BCH code is defined results in a different generator polynomial as can be seen in the example.

```
octave:1> bchpoly([1 1 0 0 1], 7)
ans =

  1  0  0  0  1  0  1  1  1

octave:2> bchpoly([1 0 0 1 1], 7)
ans =

  1  1  1  0  1  0  0  0  1
```

It is recommend not to convert the generator polynomials created by *bchpoly* into generator and parity check matrices with the BCH codes, as the underlying BCH software is faster than the generic coding software and can treat significantly longer codes.

As well as using the *encode* and *decode* functions previously discussed, the user can directly use the low-level BCH functions *bchenco* and *bchdeco*. In this case the messages must be in the format of a binary matrix with *k* columns

```
octave:1> n = 31;
octave:2> pgs = bchpoly(n);
```

```
octave:3> pg = pgs(floor(rand(1,1)*(size(pgs,1) + 1)),:); # Pick a poly
octave:4> k = pg(2);
octave:5> t = pg(3);
octave:6> msg = randint(10,k);
octave:7> code = bchenco(msg,n,k);
octave:8> noisy = code + [ones(10,1), zeros(10,n-1)];
octave:9> dec = bchdeco(code,k,t);
```

4.4 Reed-Solomon Codes

4.4.1 Representation of Reed-Solomon Messages

The Reed-Solomon coder used in this package is based on code written by Phil Karn (http://www.ka9q.net/code/fec). This code was originally written in C and has been converted for use as an octave oct-file.

Reed-Solomon codes are based on Galois Fields of even characteristics GF(2^M). Many of the properties of Galois Fields are therefore important when considering Reed-Solomon coders.

The representation of the symbols of the Reed-Solomon code differs from the other block codes, in that the other block codes use a binary representation, while the Reed-Solomon code represents each m-bit symbol by an integer. The elements of the message and codeword must be elements of the Galois Field corresponding to the Reed-Solomon code. Thus to code a message with a [7,5] Reed-Solomon code an example is

```
octave:1> m = 3;
octave:2> n = 7;
octave:3> k = 5;
octave:4> msg = gf(floor(2^m*rand(2,k)),m)
msg =
GF(2^3) array. Primitive Polynomial = D^3+D+1 (decimal 11)

Array elements =

  5  0  6  3  2
  4  1  3  1  2

octave:5> code = rsenc(msg,n,k)
code =
GF(2^3) array. Primitive Polynomial = D^3+D+1 (decimal 11)

Array elements =

  5  0  6  3  2  3  5
  4  1  3  1  2  6  3
```

The variable n is the codeword length of the Reed-Solomon coder, while k is the message length. It should be noted that k should be less than n and that $n - k$ should be even. The error correcting capability of the Reed-Solomon code is then $(n-k)/2$ symbols. m is the

number of bits per symbol, and is related to *n* by `n = 2^m - 1`. For a valid Reed-Solomon coder, *m* should be between 3 and 16.

4.4.2 Creating and Decoding Messages

The Reed-Solomon encoding function requires at least three arguments. The first *msg* is the message in encodes, the second is *n* the codeword length and *k* is the message length. Therefore *msg* must have *k* columns and the output will have *n* columns of symbols.

The message itself is many up of elements of a Galois Field GF(2^M). Normally, The order of the Galois Field (M), is related to the codeword length by `n = 2^m - 1`. Another important parameter when determining the behavior of the Reed-Solomon coder is the primitive polynomial of the Galois Field (see *gf*). Thus the messages

```
octave:1> msg0 = gf([0, 1, 2, 3],3);
octave:2> msg1 = gf([0, 1, 2, 3],3,13);
```

will not result in the same Reed-Solomon coding. Finally, the parity of the Reed-Solomon code are generated with the use of a generator polynomial. The parity symbols are then generated by treating the message to encode as a polynomial and finding the remainder of the division of this polynomial by the generator polynomial. Therefore the generator polynomial must have as many roots as `n - k`. Whether the parity symbols are placed before or afterwards the message will then determine which end of the message is the most-significant term of the polynomial representing the message. The parity symbols are therefore different in these two cases. The position of the parity symbols can be chosen by specifying 'beginning' or 'end' to *rsenc* and *rsdec*. By default the parity symbols are placed after the message.

Valid generator polynomials can be constructed with the *rsgenpoly* function. The roots of the generator polynomial are then defined by

$$g = (x - A^{bs})(x - A^{(b+1)s}) \cdots (x - A^{(b+2t-1)s}).$$

where *t* is `(n-k)/2`, A is the primitive element of the Galois Field, *b* is the first consecutive root, and *s* is the step between roots. Generator polynomial of this form are constructed by *rsgenpoly* and can be passed to both *rsenc* and *rsdec*. It is also possible to pass the *b* and *s* values directly to *rsenc* and *rsdec*. In the case of *rsdec* passing *b* and *s* can make the decoding faster.

Consider the example below.

```
octave:1> m = 8;
octave:2> n = 2^m - 1;
octave:3> k = 223;
octave:4> prim = 391;
octave:5> b = 112;
octave:6> s = 11;
octave:7> gg = rsgenpoly(n, k, prim, b, s);
octave:8> msg = gf(floor(2^m*rand(17,k)), m, prim);
octave:9> code = rsenc(msg, n, k, gg);
octave:10> noisy = code + [toeplitz([ones(1,17)], ...
 zeros(1,17)), zeros(17,238)];
```

```
octave:11> [dec, nerr] = rsdec(msg, n, k, b, s);
octave:13> nerr'
ans =

   1   2   3   4   5   6   7   8   9  10  11  12  13  14  15  16  -1

octave:12> any(msg' != dec')
ans =

  0  0  0  0  0  0  0  0  0  0  0  0  0  0  0  0  1
```

This is an interesting example in that it demonstrates many of the additional arguments of the Reed-Solomon functions. In particular this example approximates the CCSDS standard Reed-Solomon coder, lacking only the dual-basis lookup tables used in this standard. The CCSDS uses non-default values to all of the basic functions involved in the Reed-Solomon encoding, since it has a non-default primitive polynomial, generator polynomial, etc.

The example creates 17 message blocks and adds between 1 and 17 error symbols to these block. As can be seen *nerr* gives the number of errors corrected. In the case of 17 introduced errors *nerr* equals -1, indicating a decoding failure. This is normal as the correction ability of this code is up to 16 error symbols. Comparing the input message and the decoding it can be seen that as expected, only the case of 17 errors has not been correctly decoded.

4.4.3 Shortened Reed-Solomon Codes

In general the codeword length of the Reed-Solomon coder is chosen so that it is related directly to the order of the Galois Field by the formula n = 2^m = 1. Although, the underlying Reed-Solomon coding must operate over valid codeword length, there are sometimes reasons to assume the the codeword length will be shorter. In this case the message is padded with zeros before coding, and the zeros are stripped from the returned block. For example consider the shortened [6,4] Reed-Solomon below

```
octave:1> m = 3;
octave:2> n = 6;
octave:3> k = 4;
octave:4> msg = gf(floor(2^m*rand(2,k)),m)
msg =
GF(2^3) array. Primitive Polynomial = D^3+D+1 (decimal 11)

Array elements =

  7  0  2  5
  1  5  7  1

octave:5> code = rsenc(msg,n,k)
code =
GF(2^3) array. Primitive Polynomial = D^3+D+1 (decimal 11)

Array elements =
```

```
7 0 2 5 2 3
1 5 7 1 0 2
```

5 Convolutional Coding

To be written.

6 Modulations

To be written.

Currently have functions amodce, ademodce, apkconst, demodmap, modmap, qaskdeco, qaskenco, genqammod, pamdemod, pammod, pskdemod and pskmod.

7 Special Filters

To be written.

8 Galois Fields

8.1 Galois Field Basics

A Galois Field is a finite algebraic field. This package implements a Galois Field type in Octave having 2^M members where M is an integer between 1 and 16. Such fields are denoted as GF(2^M) and are used in error correcting codes in communications systems. Galois Fields having odd numbers of elements are not implemented.

The *primitive element* of a Galois Field has the property that all elements of the Galois Field can be represented as a power of this element. The *primitive polynomial* is the minimum polynomial of some primitive element in GF(2^M) and is irreducible and of order M. This means that the primitive element is a root of the primitive polynomial.

The elements of the Galois Field GF(2^M) are represented as the values 0 to 2^M -1 by Octave. The first two elements represent the zero and unity values of the Galois Field and are unique in all fields. The element represented by 2 is the primitive element of the field and all elements can be represented as combinations of the primitive element and unity as follows

Integer	Binary	Element of GF(2^M)
0	000	`0`
1	001	`1`
2	010	`A`
3	011	`A + 1`
4	100	`A^2`
5	101	`A^2 + 1`
6	110	`A^2 + A`
7	111	`A^2 + A + 1`

It should be noted that there is often more than a single primitive polynomial of GF(2^M). Each Galois Field over a different primitive polynomial represents a different realization of the Field. The representations above however rest valid.

This code was written as a challenge by Paul Kienzle (octave forge) to convert a Reed-Solomon coder I had in octave to be compatible with Matlab communications toolbox R13. This forced the need to have a complete library of functions over the even Galois Fields. Although this code was written to be compatible with the equivalent Matlab code, I did not have access to a version of Matlab with R13 installed, and thus this code is based on Matlab documentation only. No compatibility testing has been performed and so I am most interested in comments about compatibility at the e-mail address dbateman@free.fr.

8.1.1 Creating Galois Fields

To work with a Galois Field GF(2^M) in Octave, you must first create a variable that Octave recognizes as a Galois Field. This is done with the function `gf(`*a*`,`*m*`)` as follows.

```
octave:1> a = [0:7];
octave:2> b = gf(a,4)
b =
GF(2^4) array. Primitive Polynomial = D^4+D+1 (decimal 19)
```

```
Array elements =

  0   1   2   3   4   5   6   7
```

This creates an array *b* with 8 elements that Octave recognizes as a Galois Field. The field is created with the default primitive polynomial for the field GF(2^4). It can be verified that a variable is in fact a Galois Field with the functions `isgalois` or `whos`.

```
octave:3> isgalois(a)
ans = 0
octave:4> isgalois(b)
ans = 1
octave:5> whos

*** local user variables:

prot  type                        rows   cols  name
====  ====                        ====   ====  ====
 rwd  matrix                         1      8  a
 rwd  galois                         1      8  b
```

It is also possible to create a Galois Field with an arbitrary primitive polynomial. However, if the polynomial is not a primitive polynomial of the field, and error message is returned. For instance.

```
octave:1> a = [0:7];
octave:2> b = gf(a,4,25)
b =
GF(2^4) array. Primitive Polynomial = D^4+D^3+1 (decimal 25)

Array elements =

  0   1   2   3   4   5   6   7

octave:3> c = gf(a,4,21)
error: primitive polynomial (21) of Galois Field must be irreducible
error: unable to initialize Galois Field
error: evaluating assignment expression near line 3, column 3
```

The function *gftable* is included for compatibility with Matlab. In Matlab this function is used to create the lookup tables used to accelerate the computations over the Galois Field and store them to a file. However octave stores these parameters for all of the fields currently in use and so this function is not required, although it is silently accepted.

8.1.2 Primitive Polynomials

The function `gf(`*a*`,`*m*`)` creates a Galois Field using the default primitive polynomial. However there exists many possible primitive polynomials for most Galois Fields. Two functions exist for identifying primitive polynomials, *isprimitive* and *primpoly*. `primpoly(`*m*`,`*opt*`)` is used to identify the primitive polynomials of the fields GF(2^M). For example

```
octave:1> primpoly(4)

Primitive polynomial(s) =

D^4+D+1

ans = 19
```

identifies the default primitive polynomials of the field GF(2^M), which is the same as `primpoly(4,"min")`. All of the primitive polynomials of a field can be identified with the function `primpoly(`*m*`,"all")`. For example

```
octave:1> primpoly(4, "all")

Primitive polynomial(s) =

D^4+D+1
D^4+D^3+1

ans =

  19  25
```

while `primpoly(`*m*`,"max")` returns the maximum primitive polynomial of the field, which for the case above is 25. The function *primpoly* can also be used to identify the primitive polynomials having only a certain number of non-zero terms. For instance

```
octave:1> primpoly(5, 3)

Primitive polynomial(s) =

D^5+D^2+1
D^5+D^3+1

ans =

  37  41
```

identifies the polynomials with only three terms that can be used as primitive polynomials of GF(2^5). If no primitive polynomials existing having the requested number of terms then *primpoly* returns an empty vector. That is

```
octave:1> primpoly(5,2)
primpoly: No primitive polynomial satisfies the given constraints

ans = [](1x0)
```

As can be seen above, *primpoly* displays the polynomial forms the the polynomials that it finds. This output can be suppressed with the 'nodisplay' option, while the returned value is left unchanged.

```
octave:1> primpoly(4,"all","nodisplay")
ans =

  19  25
```

`isprimitive(a)` identifies whether the elements of *a* can be used as primitive polynomials of the Galois Fields GF(2^M). Consider as an example the fields GF(2^4). The primitive polynomials of these fields must have an order m and so their integer representation must be between 16 and 31. Therefore *isprimitive* can be used in a similar manner to *primpoly* as follows

```
octave:1> find(isprimitive(16:31)) + 15
ans =

  19  25
```

which finds all of the primitive polynomials of GF(2^4).

8.1.3 Accessing Internal Fields

Once a variable has been defined as a Galois Field, the parameters of the field of this structure can be obtained by adding a suffix to the variable. Valid suffixes are '.m', '.prim_poly' and '.x', which return the order of the Galois Field, its primitive polynomial and the data the variable contains respectively. For instance

```
octave:1> a = [0:7];
octave:2> b = gf(a,4);
octave:3> b.m
ans = 4
octave:4> b.prim_poly
ans = 19
octave:5> c = b.x;
octave:6> whos

*** local user variables:

prot  type                       rows   cols  name
====  ====                       ====   ====  ====
 rwd  matrix                        1      8  a
 rwd  galois                        1      8  b
 rwd  matrix                        1      8  c
```

Please note that it is explicitly forbidden to modify the galois field by accessing these variables. For instance

```
octave:1> a = gf([0:7],3);
octave:2> a.prim_poly = 13;
```

is explicitly forbidden. The result of this will be to replace the Galois array *a* with a structure *a* with a single element called '.prim_poly'. To modify the order or primitive polynomial of a field, a new field must be created and the data copied. That is

```
octave:1> a = gf([0:7],3);
octave:2> a = gf(a.x,a.m,13);
```

8.1.4 Function Overloading

An important consideration in the use of the Galois Field package is that many of the internal functions of Octave, such as *roots*, can not accept Galois Fields as an input. This

package therefore uses the *dispatch* function of Octave-Forge to *overload* the internal Octave functions with equivalent functions that work with Galois Fields, so that the standard function names can be used. However, at any time the Galois field specific version of the function can be used by explicitly calling its function name. The correspondence between the internal function names and the Galois Field versions is as follows

`conv`	-	`gconv`,	`convmtx`	-	`gconvmtx`,	`diag`	-	`gdiag`,
`deconv`	-	`gdeconv`,	`det`	-	`gdet`,	`exp`	-	`gexp`,
`filter`	-	`gfilter`,	`inv`	-	`ginv`,	`log`	-	`glog`,
`lu`	-	`glu`,	`prod`	-	`gprod`,	`reshape`	-	`greshape`,
`rank`	-	`grank`,	`roots`	-	`groots`,	`sum`	-	`gsum`,
`sumsq`	-	`gsumsq`.						

The version of the function that is chosen is determined by the first argument of the function. So, considering the *filter* function, if the first argument is a *Matrix*, then the normal version of the function is called regardless of whether the other arguments of the function are Galois vectors or not.

Other Octave functions work correctly with Galois Fields and so overloaded versions are not necessary. This include such functions as *size* and *polyval*.

It is also useful to use the '.x' option discussed in the previous section, to extract the raw data of the Galois field for use with some functions. An example is

```
octave:1> a = minpol(gf(14,5));
octave:2> b = de2bi(a.x,"left-msb");
```

converts the polynomial form of the minimum polynomial of 14 in GF(2^5) into an integer.

8.1.5 Known Problems

Before reporting a bug compare it to this list of known problems

Concatenation

For versions of Octave prior to 2.1.58, the concatenation of Galois arrays returns a Matrix type. That is `[gf([1, 0],m) gf(1, m)]` returns a matrix went it should return another Galois array. The workaround is to explicitly convert the returned value back to the correct Galois field using `gf([gf([1, 0],m) gf(1,m)],m)`.

Since Octave version 2.1.58, `[gf([1, 0],m) gf(1, m)]` returns another Galois array as expected.

Saving and loading Galois variables

Saving of Galois variables is only implemented in versions of octave later than 2.1.53. If you are using a recent version of octave then saving a Galois variable is as simple as

```
octave:2> save a.mat a
```

where *a* is a Galois variable. To reload the variable within octave, the Galois type must be installed prior to a call to *load*. That is

```
octave:1> dummy = gf(1);
octave:2> load a.mat
```

With versions of octave later than 2.1.53, Galois variables can be saved in the octave binary and ascii formats, as well as the HDF5 format. If you are using an earlier version of octave, you can not directly save a Galois variable. You can however save the information it contains and reconstruct the data afterwards by doing something like

```
octave:2> x = a.x; m = a.m; p = a.prim_poly;
octave:3> save a.mat x m p;
```

Logarithm of zero does not return NaN

The logarithm of zero in a Galois field is not defined. However, to avoid segmentation faults in later calculations the logarithm of zero is defined as `2^m-1`, whose value is not the logarithm of any other value in the Galois field. A warning is however printed to tell the user about the problem. For example

```
octave:1> m = 3;
octave:2> a = log(gf([0:2^m-1],m))
warning: log of zero undefined in Galois field
a =
GF(2^3) array. Primitive Polynomial = D^3+D+1 (decimal 11)

Array elements =

  7  0  1  3  2  6  4  5
```

To fix this problem would require a major rewrite of all code, adding an exception for the case of NaN to all basic operators. These exceptions will certainly slow the code down.

Speed

The code was written piece-meal with no attention to optimum code. Now that I have something working I should probably go back and tidy the code up, optimizing it at the same time.

8.2 Manipulating Galois Fields

8.2.1 Expressions, manipulation and assignment

Galois variables can be treated in similar manner to other variables within Octave. For instance Galois fields can be accessed using index expressions in a similar manner to all other Octave matrices. For example

```
octave:1> a = gf([[0:7];[7:-1:0]],3)
a =
GF(2^3) array. Primitive Polynomial = D^3+D+1 (decimal 11)

Array elements =

  0  1  2  3  4  5  6  7
  7  6  5  4  3  2  1  0
```

```
octave:2> b = a(1,:)
b =
GF(2^3) array. Primitive Polynomial = D^3+D+1 (decimal 11)

Array elements =

  0  1  2  3  4  5  6  7
```

Galois arrays can equally use indexed assignments. That is, the data in the array can be partially replaced, on the condition that the two fields are identical. An example is

```
octave:1> a = gf(ones(2,8),3);
octave:2> b = gf(zeros(1,8),3);
octave:3> a(1,:) = b
a =
GF(2^3) array. Primitive Polynomial = D^3+D+1 (decimal 11)

Array elements =

  0  0  0  0  0  0  0  0
  1  1  1  1  1  1  1  1
```

Implicit conversions between normal matrices and Galois arrays are possible. For instance data can be directly copied from a Galois array to a real matrix as follows.

```
octave:1> a = gf(ones(2,8),3);
octave:2> b = zeros(2,8);
octave:3> b(2,:) = a(2,:)
b =

  0  0  0  0  0  0  0  0
  1  1  1  1  1  1  1  1
```

The inverse is equally possible, with the proviso that the data in the matrix is valid in the Galois field. For instance

```
octave:1> a = gf([0:7],3);
octave:2> a(1) = 1;
```

is valid, while

```
octave:1> a = gf([0:7],3);
octave:2> a(1) = 8;
```

is not, since 8 is not an element of GF(2^3). This is a basic rule of manipulating Galois arrays. That is matrices and scalars can be used in conjunction with a Galois array as long as they contain valid data within the Galois field. In this case they will be assumed to be of the same field.

As Octave supports concatenation of typed matrices only for version 2.1.58 and later, matrix concatenation will force the Galois array back to a normal matrix for earlier version. For instance for Octave 2.1.58 and later.

```
octave:1> a = [gf([0:7],3); gf([7:-1:0],3)];
octave:2> b = [a, a];
octave:3> whos
```

```
*** local user variables:

  Prot Name        Size  Bytes  Class
  ==== ====        ====  =====  =====
   rwd a           2x8      64  galois
   rwd b           2x16    128  galois

Total is 49 elements using 192 bytes
```

and for previous versions of Octave

```
octave:1> a = [gf([0:7],3); gf([7:-1:0],3)];
octave:2> b = [a, a];
octave:3> whos

*** local user variables:

prot  type                       rows   cols  name
====  ====                       ====   ====  ====
 rwd  matrix                        2      8  a
 rwd  matrix                        2     16  b
```

This has the implication that many of the scripts included with Octave that should work with Galois fields, won't work correctly for versions earlier than 2.1.58. If you wish to concatenate Galois arrays with earlier versions, use the syntax

```
octave:1> a = gf([0:7],3);
octave:2> b = gf([a, a], a.m, a.prim_poly);
```

which explicitly reconverts *b* to the correct Galois Field. Other basic manipulations of Galois arrays are

`isempty` Returns true if the Galois array is empty.

`size` Returns the number of rows and columns in the Galois array.

`length` Returns the length of a Galois vector, or the maximum of rows or columns of Galois arrays.

`find` Find the indexes of the non-zero elements of a Galois array.

`diag` Create a diagonal Galois array from a Galois vector, or extract a diagonal from a Galois array.

`reshape` Change the shape of the Galois array.

8.2.2 Unary operations

The same unary operators that are available for normal Octave matrices are also available for Galois arrays. These operations are

`+x` Unary plus. This operator has no effect on the operand.

`-x` Unary minus. Note that in a Galois Field this operator also has no effect on the operand.

`!x` Returns true for zero elements of Galois Array.

`x'` Complex conjugate transpose. As the Galois Field only contains integer values, this is equivalent to the transpose operator.

`x.'` Transpose of the Galois array.

8.2.3 Arithmetic operations

The available arithmetic operations on Galois arrays are the same as on other Octave matrices. It should be noted that both operands must be in the same Galois Field. If one operand is a Galois array and the second is a matrix or scalar, then the second operand is silently converted to the same Galois Field. The element(s) of these matrix or scalar must however be valid members of the Galois field. Thus

```
octave:1> a = gf([0:7],3);
octave:2> b = a + [0:7];
```

is valid, while

```
octave:1> a = gf([0:7],3);
octave:2> b = a + [1:8];
```

is not, since 8 is not a valid element of GF(2^3). The available arithmetic operators are

`x + y` Addition. If both operands are Galois arrays or matrices, the number of rows and columns must both agree. If one operand is a is a Galois array with a single element or a scalar, its value is added to all the elements of the other operand. The `+` operator on a Galois Field is equivalent to an exclusive-or on normal integers.

`x .+ y` Element by element addition. This operator is equivalent to `+`.

`x - y` As both `+` and `-` in a Galois Field are equivalent to an exclusive-or for normal integers, `-` is equivalent to the `+` operator

`x .- y` Element by element subtraction. This operator is equivalent to `-`.

`x * y` Matrix multiplication. The number of columns of *x* must agree with the number of rows of *y*.

`x .* y` Element by element multiplication. If both operands are matrices, the number of rows and columns must both agree.

`x / y` Right division. This is conceptually equivalent to the expression

```
(inverse (y') * x')'
```

but it is computed without forming the inverse of *y'*.

If the matrix is singular then an error occurs. If the matrix is under-determined, then a particular solution is found (but not minimum norm). If the solution is over-determined, then an attempt is made to find a solution, but this is not guaranteed to work.

`x ./ y` Element by element right division.

`x \ y` Left division. This is conceptually equivalent to the expression

```
inverse (x) * y
```

but it is computed without forming the inverse of *x*.

If the matrix is singular then an error occurs. If the matrix is under-determined, then a particular solution is found (but not minimum norm). If the solution is over-determined, then an attempt is made to find a solution, but this is not guaranteed to work.

x `.\` *y* Element by element left division. Each element of *y* is divided by each corresponding element of *x*.

x `^` *y*
x `**` *y* Power operator. If *x* and *y* are both scalars, this operator returns *x* raised to the power *y*. Otherwise *x* must be a square matrix raised to an integer power.

x `.^` *y*
x `.**` *y* Element by element power operator. If both operands are matrices, the number of rows and columns must both agree.

8.2.4 Comparison operations

Galois variables can be tested for equality in the usual manner. That is

```
octave:1> a = gf([0:7],3);
octave:2> a == ones(1,8)
ans =

  0  1  0  0  0  0  0  0

octave:3> a ~= zeros(1,8)
ans =

  0  1  1  1  1  1  1  1
```

Likewise, Galois vectors can be tested against scalar values (whether they are Galois or not). For instance

```
octave:4> a == 1
ans =

  0  1  0  0  0  0  0  0
```

To test if any or all of the values in a Galois array are non-zero, the functions *any* and *all* can be used as normally.

In addition the comparison operators `>`, `>=`, `<` and `<=` are available. As elements of the Galois Field are modulus 2^*m*, all elements of the field are both greater than and less than all others at the same time.Thus these comparison operators don't make that much sense and are only included for completeness. The comparison is done relative to the integer value of the Galois Field elements.

8.2.5 Polynomial manipulations

A polynomial in GF(2^M) can be expressed as a vector in GF(2^M). For instance if *a* is the *primitive element*, then the example

```
octave:1> poly = gf([2, 4, 5, 1],3);
```

represents the polynomial

$$poly = ax^3 + a^2x^2 + (a^2 + 1)x + 1$$

Arithmetic can then be performed on these vectors. For instance to add to polynomials an example is

```
octave:1> poly1 = gf([2, 4, 5, 1],3);
octave:2> poly2 = gf([1, 2],3);
octave:3> sumpoly = poly1 + [0, 0, poly2]
sumpoly =
GF(2^3) array. Primitive Polynomial = D^3+D+1 (decimal 11)

Array elements =

  2  4  4  3
```

Note that *poly2* must be zero padded to the same length as poly1 to allow the addition to take place.

Multiplication and division of Galois polynomials is equivalent to convolution and de-convolution of vectors of Galois elements. Thus to multiply two polynomials in GF(2^3).

```
octave:4> mulpoly = conv(poly1, poly2)
mulpoly =
GF(2^3) array. Primitive Polynomial = D^3+D+1 (decimal 11)

Array elements =

  2  0  6  0  2
```

Likewise the division of two polynomials uses the de-convolution function as follows

```
octave:5> [poly, remd] = deconv(mulpoly,poly2)
poly =
GF(2^3) array. Primitive Polynomial = D^3+D+1 (decimal 11)

Array elements =

  2  4  5  1

remd =
GF(2^3) array. Primitive Polynomial = D^3+D+1 (decimal 11)

Array elements =

  0  0  0  0  0
```

Note that the remainder of this division is zero, as we performed the inverse operation to the multiplication.

To evaluate a polynomial for a certain value in GF(2^M), use the Octave function *polyval*.

```
octave:1> poly1 = gf([2, 4, 5, 1],3);  ## a*x^3+a^2*x^2+(a^2+1)*x+1
octave:2> x0 = gf([0, 1, 2],3);
octave:3> y0 = polyval(poly1, x0);
y0 =
GF(2^3) array. Primitive Polynomial = D^3+D+1 (decimal 11)

Array elements =

  1  2  0

octave:4> a = gf(2,3);               ## The primitive element
octave:5> y1 = a .* x0.^3 + a.^2 .* x0.^2 + (a.^2 + 1) .* x0 + 1
y1 =
GF(2^3) array. Primitive Polynomial = D^3+D+1 (decimal 11)

Array elements =

  1  2  0
```

It is equally possible to find the roots of Galois polynomials with the *roots* function. Using the polynomial above over GF(2^3), we can find its roots in the following manner

```
octave:1> poly1 = gf([2, 4, 5, 1], 3);
octave:2> root1 = roots(poly1)
root1 =
GF(2^3) array. Primitive Polynomial = D^3+D+1 (decimal 11)

Array elements =

  2
  5
  5
```

Thus the example polynomial has 3 roots in GF(2^3) with one root of multiplicity 2. We can check this answer with the *polyval* function as follows

```
octave:3> check1 = polyval(poly1, root1)
check1 =
GF(2^3) array. Primitive Polynomial = D^3+D+1 (decimal 11)

Array elements =

  0
  0
  0
```

which as expected gives a zero vector. It should be noted that both the number of roots and their value, will depend on the chosen field. Thus for instance

```
octave:1> poly3 = gf([2, 4, 5, 1],3, 13);
octave:2> root3 = roots(poly3)
root3 =
GF(2^3) array. Primitive Polynomial = D^3+D^2+1 (decimal 13)
```

```
Array elements =

  5
```

shows that in the field GF(2^3) with a different primitive polynomial, has only one root exists.

The minimum polynomial of an element of GF(2^M) is the minimum degree polynomial in GF(2), excluding the trivial zero polynomial, that has that element as a root. The fact that the minimum polynomial is in GF(2) means that its coefficients are one or zero only. The *minpol* function can be used to find the minimum polynomial as follows

```
octave:1> a = gf(2,3);                ## The primitive element
octave:2> b = minpol(a)
b =
GF(2) array.

Array elements =

  1  0  1  1
```

Note that the minimum polynomial of the primitive element is the primitive polynomial. Elements of GF(2^M) sharing the same minimum polynomial form a partitioning of the field. This partitioning can be found with the *cosets* function as follows

```
octave:1> c = cosets(3)
c =
{
  [1,1] =
  GF(2^3) array. Primitive Polynomial = D^3+D+1 (decimal 11)

  Array elements =

    1

  [2,1] =
  GF(2^3) array. Primitive Polynomial = D^3+D+1 (decimal 11)

  Array elements =

    2  4  6

  [3,1] =
  GF(2^3) array. Primitive Polynomial = D^3+D+1 (decimal 11)

  Array elements =

    3  5  7

}
```

which returns a cell array containing all of the the elements of the GF(2^3), partitioned into groups sharing the same minimum polynomial. The function *cosets* can equally ac-

cept a second argument defining the primitive polynomial to use in its calculations (i.e. `cosets(a,p)`).

8.2.6 Linear Algebra

The basic linear algebra operation of this package is the LU factorization of a the Galois array. That is the Galois array *a* is factorized in the following way

```
octave:2> [l, u, p] = lu(a)
```

such that $p * a = l * u$. The matrix *p* contains row permutations of *a*, such that *l* and *u* are strictly upper and low triangular. The Galois array *a* can be rectangular.

All other linear algebra operations within this package are based on this LU factorization of a Galois array. An important consequence of this is that no solution can be found for singular matrices, only a particular solution will be found for under-determined systems of equation and the solution found for over-determined systems is not always correct. This is identical to the way Matlab R13 treats this.

For instance consider the under-determined linear equation

```
octave:1> A = gf([2, 0, 3, 3; 3, 1, 3, 1; 3, 1, 1, 0], 2);
octave:2> b = [0:2]';
octave:3> x = A \ b;
```

gives the solution *x* = `[2, 0, 3, 2]`. There are in fact 4 possible solutions to this linear system; *x* = `[3, 2, 2, 0]`, *x* = `[0, 3, 1, 1]`, *x* = `[2, 0, 3, 2]` and *x* = `[1, 1, 0, 3]`. No particular selection criteria are applied to the chosen solution.

In addition the fact that singular matrices are not treated, unless you know the matrix is not singular, you should test the determinant of the matrix prior to solving the linear system. For instance

```
octave:1> A = gf(floor(2^m * rand(3)), 2);
octave:2> b = [0:2]';
octave:3> if (det(A) ~= 0); x = A \ b; y = b' / A; end;
octave:4> r = rank(A);
```

solves the linear systems $A * x = b$ and $y * A = b$. Note that you do not need to take into account rounding errors in the determinant, as the determinant can only take values within the Galois Field. So if the determinant equals zero, the array is singular.

8.2.7 Signal Processing with Galois Fields

Signal processing functions such as filtering, convolution, de-convolution and Fourier transforms can be performed over Galois Fields. For instance the *filter* function can be used with Galois vectors in the same manner as usual. For instance

```
octave:1> b = gf([2, 0, 0, 1, 0, 2, 0, 1],2);
octave:2> a = gf([2, 0, 1, 1],2);
octave:3> x = gf([1, zeros(1,20)],2);
octave:4> y = filter(b, a, x)
y =
GF(2^2) array. Primitive Polynomial = D^2+D+1 (decimal 7)

Array elements =
```

```
1  0  3  0  2  3  1  0  1  3  3  1  0  1  3  3  1  0  1  3  3
```

gives the impulse response of the filter defined by *a* and *b*.

Two equivalent ways are given to perform the convolution of two Galois vectors. Firstly the function *conv* can be used, or alternatively the function *convmtx* can be used. The first of these function is identical to the convolution function over real vectors, and has been described in the section about multiplying two Galois polynomials.

In the case where many Galois vectors will be convolved with the same vector, the second function *convmtx* offers an alternative method to calculate the convolution. If *a* is a column vector and *x* is a column vector of length *n*, then

```
octave:1> m = 3;
octave:2> a = gf(floor(2^m*rand(4,1)),m);
octave:3> b = gf(floor(2^m*rand(4,1)),m);
octave:4> c0 = conv(a,b)';
octave:5> c1 = convmtx(a,length(b)) * b;
octave:6> check = all(c0 == c1)
check = 1
```

shows the equivalence of the two functions. The de-convolution function has been previously described above.

The final signal processing function available in this package are the functions to perform Fourier transforms over a Galois field. Three functions are available, *fft*, *ifft* and *dftmtx*. The first two functions use the third to perform their work. Given an element *a* of the Galois field GF(2^M), *dftmtx* returns the `2^M - 1` square matrix used in the Fourier transforms with respect to *a*. The minimum polynomial of *a* must be primitive in GF(2^M). In the case of the *fft* function *dftmtx* is called with the the primitive element of the Galois Field as an argument. As an example

```
octave:1> m = 4;
octave:2> n = 2^m -1;
octave:2> alph = gf(2, m);
octave:3> x = gf(floor(2^m*rand(n,1)), m);
octave:4> y0 = fft(x);
octave:5> y1 = dftmtx(alph) * x;
octave:6> z0 = ifft(y0);
octave:7> z1 = dftmtx(1/alph) * y1;
octave:8> check = all(y0 == y1) & all(z0 == x) & all(z1 == x)
check = 1
```

In all cases, the length of the vector to be transformed must be `2^M -1`. As the *dftmtx* creates a matrix representing the Fourier transform, to limit the computational task only Fourier transforms in GF(2^M), where M is less than or equal to 8, can be treated.

9 Function Reference

9.1 Functions by Category

9.1.1 Random Signals

awgn Add white Gaussian noise to a voltage signal

biterr Compares two matrices and returns the number of bit errors and the bit error rate.

eyediagram
Plot the eye-diagram of a signal.

randerr Generate a matrix of random bit errors.

randint Generate a matrix of random binary numbers.

randsrc Generate a matrix of random symbols.

scatterplot
Display the scatter plot of a signal.

symerr Compares two matrices and returns the number of symbol errors and the symbol error rate.

wgn Returns a M-by-N matrix Y of white Gaussian noise.

bsc Send DATA into a binary symetric channel with probability P of error one each symbol

9.1.2 Source Coding

arithenco *Not implemented*

arithdeco *Not implemented*

compand Compresses and expanding the dynamic range of a signal using a mu-law or or A-law algorithm

dpcmdeco *Not implemented*

dpcmenco *Not implemented*

dpcmopt *Not implemented*

huffmandeco
Returns the original signal that was Huffman encoded signal using 'huffmanenco'.

huffmandict
Builds a Huffman code, given a probability list.

huffmanenco
Returns the Huffman encoded signal using DICT.

lloyds — Optimize the quantization table and codes to reduce distortion.

lz77deco — Lempel-Ziv 77 source algorithm decoding implementation.

lz77enco — Lempel-Ziv 77 source algorithm implementation.

quantiz — Quantization of an arbitrary signal relative to a paritioning

shannonfanodict
: Returns the code dictionary for source using shanno fano algorithm Dictionary is built from SYMBOL_PROBABILITIES using the shannon fano scheme.

shannonfanoenco
: Returns the Shannon Fano encoded signal using DICT This function uses a DICT built from the 'shannonfanodict' and uses it to encode a signal list into a shannon fano code Restrictions include a signal set that strictly belongs in the 'range [1,N]' with 'N=length(dict)'.

shannonfanodeco
: Returns the original signal that was Shannonfano encoded.

rleenco — Returns run-length encoded MESSAGE.

rledeco — Returns decoded run-length MESSAGE The RLE encoded MESSAGE has to be in the form of a row-vector.

riceenco — Returns the Rice encoded signal using K or optimal K Default optimal K is chosen between 0-7.

ricedeco — Returns the Rice decoded signal vector using CODE and K Compulsory K is need to be specified A restrictions is that a signal set must strictly be non-negative The value of code is a cell array of row-vectors which have the encoded rice value for a single sample.

fiboenco — Returns the cell-array of encoded fibonacci value from the column vectors NUM Universal codes like fibonacci codes have a useful synchronization property, only for 255 maximum value we have designed these routines.

fibodeco — Returns the decoded fibonacci value from the binary vectors CODE Universal codes like fibonacci codes Have a useful synchronization property, only for 255 maximum value we have designed these routines.

fibosplitstream
: Returns the split data stream at the word boundaries Assuming the stream was originally encoded using 'fiboenco' and this routine splits the stream at the points where '11' occur together & gives us the code-words which can later be decoded from the 'fibodeco' This however doesnt mean that we intend to verify if all the codewords are correct, and infact the last symbol in th return list can or can-not be a valid codeword

golombenco
: Returns the Golomb coded signal as cell array Also total length of output code in bits can be obtained This function uses a M need to be supplied for encoding signal vector into a golomb coded vector.

golombdeco
: Returns the Golomb decoded signal vector using CODE and M Compulsory m is need to be specified.

9.1.3 Block Interleavers

intrlv
: Interleaved elements of DATA according to ELEMENTS See also: deintrlv

algintrlv
: *Not implemented*

helscanintrlv
: *Not implemented*

matintrlv
: Interleaved elements of DATA with a tempory matrix of size NROWS-by-NCOLS See also: matdeintrlv

randintrlv
: Interleaves elements of DATA with a random permutation See also: intrlv,deintrlv

deintrlv
: Restore elements of DATA according to ELEMENTS See also: intrlv

matdeintrlv
: Restore elements of DATA with a tempory matrix of size NROWS-by-NCOLS See also: matintrlv

randdeintrlv
: Restore elements of DATA with a random permutation See also: randintrlv,intrlv,deintrlv

9.1.4 Block Coding

bchdeco
: Decodes the coded message CODE using a BCH coder.

bchenco
: Encodes the message MSG using a [N,K] BCH coding.

bchpoly
: Calculates the generator polynomials for a BCH coder.

convenc
: *Not implemented*

cyclgen
: Produce the parity check and generator matrix of a cyclic code.

cyclpoly
: This function returns the cyclic generator polynomials of the code [N,K].

decode
: Top level block decoder.

encode
: Top level block encoder.

egolaydec
: Given R, the received Extended Golay code, this function tries to decode R using the Extended Golay code parity check matrix Extended Golay code (24,12) which can correct upto 3 errors

egolayenc
: Given M, encode M using the Extended Golay code

egolaygen
: Returns the Extended Golay code (24,12) generator matrix, which can correct upto 3 errors.

gen2par
: Converts binary generator matrix GEN to the parity chack matrix PAR and visa-versa.

hammgen Produce the parity check and generator matrices of a Hamming code.

reedmullerdec
Decode the received code word VV using the RM-generator matrix G, of order R, M, returning the code-word C.

reedmullerenc
Definition type construction of Reed Muller code, of order R, length 2^M.

reedmullergen
Definition type construction of Reed Muller code, of order R, length 2^M.

rsgenpoly Creates a generator polynomial for a Reed-Solomon coding with message length of K and codelength of N.

rsdec Decodes the message contained in CODE using a [N,K] Reed-Solomon code.

rsdecof Decodes an ascii file using a Reed-Solomon coder.

rsenc Encodes the message MSG using a [N,K] Reed-Solomon coding.

rsencof Encodes an ascii file using a Reed-Solomon coder.

systematize
Given G, extract P partiy check matrix.

syndtable Create the syndrome decoding table from the parity check matrix H.

vitdec *Not implemented*

9.1.5 Modulations

ademod *Not implemented*

ademodce Baseband demodulator for analog signals.

amod *Not implemented*

amodce Baseband modulator for analog signals.

ammod Create the AM modulation of the signal x with carrier frequency fs.

amdemod Compute the amplitude demodulation of the signal S with a carrier frequency of FC and a sample frequency of FS See also: ammod

apkconst Plots a ASK/PSK signal constellation.

ddemod *Not implemented*

ddemodce *Not implemented*

demodmap
Demapping of an analog signal to a digital signal.

dmod *Not implemented*

dmodce *Not implemented*

fmmod Create the FM modulation of the signal x with carrier frequency fs.

fmdemod Create the FM demodulation of the signal x with carrier frequency fs.

genqammod
Modulates an information sequence of intergers X in the range '[0 ... M-1]' onto a quadrature amplitude modulated signal Y, where 'M = length(c) - 1' and C is a 1D vector specifing the signal constellation mapping to be used.

genqamdemod
General quadrature amplitude demodulation.

modmap Mapping of a digital signal to an analog signal.

pamdemod
Demodulates a pulse amplitude modulated signal X into an information sequence of integers in the range '[0 ... M-1]' PHI controls the initial phase and TYPE controls the constellation mapping.

pammod Modulates an information sequence of integers X in the range '[0 ... M-1]' onto a pulse amplitude modulated signal Y PHI controls the initial phase and TYPE controls the constellation mapping.

pskdemod Demodulates a complex-baseband phase shift keying modulated signal into an information sequence of integers in the range '[0 ... M-1]'.

pskmod Modulates an information sequence of integers X in the range '[0 ... M-1]' onto a complex baseband phase shift keying modulated signal Y.

qaskdeco Demaps an analog signal using a square QASK constellation.

qaskenco Map a digital signal using a square QASK constellation.

qammod Create the QAM modulation of x with a size of alphabet m See also: qamdemod,pskmod,pskdemod

qamdemod
Create the QAM demodulation of x with a size of alphabet m See also: qammod,pskmod,pskdemod

9.1.6 Special Filters

hank2sys *Not implemented*

hilbiir *Not implemented*

rcosflt *Not implemented*

rcosiir *Not implemented*

rcosine *Not implemented*

rcosfir *Not implemented*

9.1.7 Galois Fields of Even Characateristic

+ -	Addition and subtraction in a Galois Field.
* / \	Matrix multiplication and division of Galois arrays.
.* ./ .\	Element by element multiplication and division of Galois arrays.
** ^	Matrix exponentiation of Galois arrays.
.** .^	Element by element matrix exponentiation of Galois arrays.
' .'	Matrix transpose of Galois arrays.
== ~= != > >= < <=	Logical operators on Galois arrays.
all	*Not implemented*
any	*Not implemented*
cosets	Finds the elements of GF(2^M) with primitive polynomial PRIM, that share the same minimum polynomial.
gconv	Convolve two Galois vectors
gconvmtx	Create matrix to perform repeated convolutions with the same vector in a Galois Field.
gdeconv	Deconvolve two Galois vectors
gdet	Compute the determinant of the Galois array A.
gdftmtx	Form a matrix, that can be used to perform Fourier transforms in a Galois Field
gdiag	Return a diagonal matrix with Galois vector V on diagonal K.
gexp	Compute the anti-logarithm for each element of X for a Galois array.
gf	Creates a Galois field array GF(2^M) from the matrix X.
gfft	If X is a column vector, finds the FFT over the primitive element of the Galois Field of X.
gfilter	Digital filtering of vectors in a Galois Field.
gftable	This function exists for compatiability with matlab.
gfweight	Calculate the minimum weight or distance of a linear block code.
gifft	If X is a column vector, finds the IFFT over the primitive element of the Galois Field of X.
ginv	Compute the inverse of the square matrix A.
ginverse	See ginv.
gisequal	Return true if all of X1, X2, ... are equal See also: isequalwithequalnans
glog	Compute the natural logarithm for each element of X for a Galois array.
glu	Compute the LU decomposition of A in a Galois Field.

gprod Product of elements along dimension DIM of Galois array.

gsqrt Compute the square root of X, element by element, in a Galois Field.

grank Compute the rank of the Galois array A by counting the independent rows and columns.

greshape Return a matrix with M rows and N columns whose elements are taken from the Galois array A.

groots For a vector V with N components, return the roots of the polynomial over a Galois Field

gsum Sum of elements along dimension DIM of Galois array.

gsumsq Sum of squares of elements along dimension DIM of Galois array.

isempty *Not implemented*

isgalois Return 1 if the value of the expression EXPR is a Galois Field.

isprimitive Returns 1 is the polynomial represented by A is a primitive polynomial of GF(2).

length *Not implemented*

minpol Finds the minimum polynomial for elements of a Galois Field.

polyval *Not implemented*

primpoly Finds the primitive polynomials in GF(2^M).

size *Not implemented*

9.1.8 Galois Fields of Odd Characteristic

gfadd *Not implemented*

gfconv *Not implemented*

gfcosets *Not implemented*

gfdeconv *Not implemented*

gfdiv *Not implemented*

gffilter *Not implemented*

gflineq *Not implemented*

gfminpol *Not implemented*

gfmul *Not implemented*

gfpretty *Not implemented*

gfprimck *Not implemented*

gfprimdf *Not implemented*

gfprimfd *Not implemented*

gfrank *Not implemented*

gfrepcov *Not implemented*

gfroots *Not implemented*

gfsub *Not implemented*

gftrunc *Not implemented*

gftuple *Not implemented*

9.1.9 Utility Functions

comms Manual and test code for the Octave Communications toolbox.

bi2de Convert bit matrix to a vector of integers

de2bi Convert a non-negative integer to bit vector

oct2dec Convert octal to decimal values See also: base2dec,bin2dec,dec2bi

istrellis *Not implemented*

poly2trellis
Not implemented

vec2mat Converts the vector V into a C column matrix with row priority arrangement and with the final column padded with the value D to the correct length.

qfunc Compute the Q function See also: erfc, erf

qfuncinv Compute the inverse Q function See also: erfc, erf

marcumq Compute the Marcum Q function

9.2 Functions Alphabetically

9.2.1 ademodce

y = **ademodce** (*x*,*Fs*,`'amdsb-tc'`,`offset`) Function File
y = **ademodce** (*x*,*Fs*,`'amdsb-tc/costas'`,`offset`) Function File
y = **ademodce** (*x*,*Fs*,`'amdsb-sc'`) Function File
y = **ademodce** (*x*,*Fs*,`'amdsb-sc/costas'`) Function File
y = **ademodce** (*x*,*Fs*,`'amssb'`) Function File
y = **ademodce** (*x*,*Fs*,`'qam'`) Function File
y = **ademodce** (*x*,*Fs*,`'qam/cmplx'`) Function File
y = **ademodce** (*x*,*Fs*,`'fm'`,*dev*) Function File
y = **ademodce** (*x*,*Fs*,`'pm'`,*dev*) Function File
y = **ademodce** (*x*,[*Fs*,*iphs*],...) Function File
y = **ademodce** (...,*num*,*den*) Function File

Baseband demodulator for analog signals. The input signal is specified by *x*, its sampling frequency by *Fs* and the type of modulation by the third argument, *typ*. The default values of *Fs* is 1 and *typ* is 'amdsb-tc'

If the argument *Fs* is a two element vector, the the first element represents the sampling rate and the second the initial phase

The different types of demodulations that are available are

'am'
'amdsb-tc' Double-sideband with carrier

'amdsb-tc/costas'
Double-sideband with carrier and Costas phase locked loop

'amdsb-sc' Double-sideband with suppressed carrier

'amssb' Single-sideband with frequency domain Hilbert filtering

'qam' Quadrature amplitude demodulation. In-phase in odd-columns and quadrature in even-columns

'qam/cmplx'
Quadrature amplitude demodulation with complex return value

'fm' Frequency demodulation

'pm' Phase demodulation

Additional arguments are available for the demodulations 'amdsb-tc', 'fm', 'pm'. These arguments are

`offset` The offset in the input signal for the transmitted carrier

`dev` The deviation of the phase and frequency modulation

It is possible to specify a low-pass filter, by the numerator *num* and denominator *den* that will be applied to the returned vector

See also: ademodce,dmodce

9.2.2 amdemod

[*m*] = **amdemod** (*s*, *fc*, *fs*) Function File

Compute the amplitude demodulation of the signal *s* with a carrier frequency of *fc* and a sample frequency of *fs* See also: ammod

9.2.3 ammod

`ammod` (*x*,*fc*,*fs*) Function File

Create the AM modulation of the signal x with carrier frequency fs. Where x is sample at frequency fs See also: amdemod,fmmod,fmdemod

9.2.4 amodce

y = **amodce** (*x*,*Fs*,`'amdsb-tc'`,`offset`) Function File
y = **amodce** (*x*,*Fs*,`'amdsb-sc'`) Function File
y = **amodce** (*x*,*Fs*,`'amssb'`) Function File
y = **amodce** (*x*,*Fs*,`'amssb/time'`,*num*,*den*) Function File
y = **amodce** (*x*,*Fs*,`'qam'`) Function File
y = **amodce** (*x*,*Fs*,`'fm'`,*dev*) Function File
y = **amodce** (*x*,*Fs*,`'pm'`,*dev*) Function File
y = **amodce** (*x*,[*Fs*,*iphs*],...) Function File

Baseband modulator for analog signals. The input signal is specified by *x*, its sampling frequency by *Fs* and the type of modulation by the third argument, *typ*. The default values of *Fs* is 1 and *typ* is 'amdsb-tc'

If the argument *Fs* is a two element vector, the the first element represents the sampling rate and the second the initial phase

The different types of modulations that are available are

'am'
'amdsb-tc' Double-sideband with carrier

'amdsb-sc' Double-sideband with suppressed carrier

'amssb' Single-sideband with frequency domain Hilbert filtering

'amssb/time'
Single-sideband with time domain filtering. Hilbert filter is used by default, but the filter can be specified

'qam' Quadrature amplitude modulation

'fm' Frequency modulation

'pm' Phase modulation

Additional arguments are available for the modulations 'amdsb-tc', 'fm, 'pm' and 'amssb/time'. These arguments are

`offset` The offset in the input signal for the transmitted carrier

`dev` The deviation of the phase and frequency modulation

`num`
`den` The numerator and denominator of the filter transfer function for the time domain filtering of the SSB modulation

See also: ademodce,dmodce

9.2.5 apkconst

apkconst (*nsig*) Function File
apkconst (*nsig*, *amp*) Function File
apkconst (*nsig*, *amp*, *phs*) Function File
apkconst (*...*, "n") Function File
apkconst (*...*, *str*) Function File
y = **apkconst** (*...*) Function File

Plots a ASK/PSK signal constellation. Argument *nsig* is a real vector whose length determines the number of ASK radii in the constellation The values of vector *nsig* determine the number of points in each ASK radii

By default the radii of each ASK modulated level is given by the index of *nsig*. The amplitudes can be defined explictly in the variable *amp*, which is a vector of the same length as *nsig*

By default the first point in each ASK radii has zero phase, and following points are coding in an anti-clockwise manner. If *phs* is defined then it is a vector of the same length as *nsig* defining the initial phase in each ASK radii

In addition *apkconst* takes two string arguments 'n' and and *str* If the string 'n' is included in the arguments, then a number is printed next to each constellation point giving the symbol value that would be mapped to this point by the *modmap* function. The argument *str* is a plot style string (example 'r+') and determines the default gnuplot point style to use for plot points in the constellation

If *apskconst* is called with a return argument, then no plot is created. However the return value is a vector giving the in-phase and quadrature values of the symbols in the constellation

See also: dmod,ddemod,modmap,demodmap

9.2.6 awgn

y = **awgn** (*x*, *snr*) Function File
y = **awgn** (*x*, *snr*, *pwr*) Function File
y = **awgn** (*x*, *snr*, *pwr*, *seed*) Function File
y = **awgn** (*...*, '*type*') Function File

Add white Gaussian noise to a voltage signal

The input *x* is assumed to be a real or complex voltage signal. The returned value *y* will be the same form and size as *x* but with Gaussian noise added. Unless the power is specified in *pwr*, the signal power is assumed to be 0dBW, and the noise of *snr* dB will be added with respect to this. If *pwr* is a numeric value then the signal *x* is assumed to be *pwr* dBW, otherwise if *pwr* is 'measured', then the power in the signal will be measured and the noise added relative to this measured power

If *seed* is specified, then the random number generator seed is initialized with this value

By default the *snr* and *pwr* are assumed to be in dB and dBW respectively. This default behaviour can be chosen with *type* set to 'dB'. In the case where *type* is set to 'linear', *pwr* is assumed to be in Watts and *snr* is a ratio

See also: randn,wgn

9.2.7 bchdeco

msg = **bchdeco** (*code*,*k*,*t*) Loadable Function
msg = **bchdeco** (*code*,*k*,*t*,*prim*) Loadable Function
msg = **bchdeco** (...,*parpos*) Loadable Function
[*msg*, *err*] = **bchdeco** (...) Loadable Function
[*msg*,*err*,*ccode*] = **bchdeco** (...) Loadable Function

Decodes the coded message *code* using a BCH coder. The message length of the coder is defined in variable *k*, and the error corerction capability of the code is defined in *t*.

The variable *code* is a binary array with *n* columns and an arbitrary number of rows. Each row of *code* represents a single symbol to be decoded by the BCH coder. The decoded message is returned in the binary array *msg* containing *k* columns and the same number of rows as *code*.

The use of *bchdeco* can be seen in the following short example.

```
m = 3; n = 2^m -1; k = 4; t = 1;
msg = randint(10,k);
code = bchenco(msg, n, k);
noisy = mod(randerr(10,n) + code,2);
[dec err] = bchdeco(msg, k, t);
```

Valid codes can be found using *bchpoly*. In general the codeword length *n* should be of the form `2^`*m*`-1`, where m is an integer. However, shortened BCH codes can be used such that if `[2^`*m*`-1,`*k*`]` is a valid code `[2^`*m*`-1-`*x*`,`*k*`-`*x*`]` is also a valid code using the same generator polynomial.

By default the BCH coding is based on the properties of the Galois Field GF(2^*m*). The primitive polynomial used in the Galois can be overridden by a primitive polynomial in *prim*. Suitable primitive polynomials can be constructed with *primpoly*. The form of *prim* maybe be either a integer representation of the primitve polynomial as given by *primpoly*, or a binary representation that might be constructed like

```
m = 3;
prim = de2bi(primpoly(m));
```

By default the parity symbols are assumed to be placed at the beginning of the coded message. The variable *parpos* controls this positioning and can take the values 'beginning' or 'end'.

See also: bchpoly,bchenco,decode,primpoly

9.2.8 bchenco

code = **bchenco** (*msg*,*n*,*k*) Loadable Function
code = **bchenco** (*msg*,*n*,*k*,*g*) Loadable Function
code = **bchenco** (...,*parpos*) Loadable Function

Encodes the message *msg* using a [*n*,*k*] BCH coding. The variable *msg* is a binary array with *k* columns and an arbitrary number of rows. Each row of *msg* represents a single symbol to be coded by the BCH coder. The coded message is returned in the binary array *code* containing *n* columns and the same number of rows as *msg*.

The use of *bchenco* can be seen in the following short example.

```
m = 3; n = 2^m -1; k = 4;
msg = randint(10,k);
code = bchenco(msg, n, k);
```

Valid codes can be found using *bchpoly*. In general the codeword length *n* should be of the form `2^m-1`, where m is an integer. However, shortened BCH codes can be used such that if `[2^m-1,k]` is a valid code `[2^m-1-x,k-x]` is also a valid code using the same generator polynomial.

By default the generator polynomial used in the BCH coding is based on the properties of the Galois Field GF(2^*m*). This default generator polynomial can be overridden by a polynomial in *g*. Suitable generator polynomials can be constructed with *bchpoly*.

By default the parity symbols are placed at the beginning of the coded message. The variable *parpos* controls this positioning and can take the values 'beginning' or 'end'.

See also: bchpoly,bchdeco,encode

9.2.9 bchpoly

p = **bchpoly** () Function File
p = **bchpoly** (*n*) Function File
p = **bchpoly** (*n*,*k*) Function File
p = **bchpoly** (*prim*,*k*) Function File
p = **bchpoly** (*n*,*k*,*prim*) Function File
p = **bchpoly** (...,*probe*) Function File
[*p*,*f*] = **bchpoly** (...) Function File
[*p*,*f*,*c*] = **bchpoly** (...) Function File
[*p*,*f*,*c*,*par*] = **bchpoly** (...) Function File
[*p*,*f*,*c*,*par*,*t*] = **bchpoly** (...) Function File

Calculates the generator polynomials for a BCH coder. Called with no input arguments *bchpoly* returns a list of all of the valid BCH codes for the codeword length 7, 15, 31, 63, 127, 255 and 511. A three column matrix is returned with each row representing a seperate valid BCH code. The first column is the codeword length, the second the message length and the third the error correction capability of the code

Called with a single input argument, *bchpoly* returns the valid BCH codes for the specified codeword length *n*. The output format is the same as above

When called with two or more arguments, *bchpoly* calculates the generator polynomial of a particular BCH code. The generator polynomial is returned in *p* as a vector representation of a polynomial in GF(2). The terms of the polynomial are listed least-significant term first

The desired BCH code can be specified by its codeword length *n* and its message length *k*. Alternatively, the primitive polynomial over which to calculate the polynomial can be specified as *prim* If a vector representation of the primitive polynomial is given, then *prim* can be specified as the first argument of two arguments, or as the third argument. However, if an integer representation of the primitive polynomial is used, then the primitive polynomial must be specified as the third argument

When called with two or more arguments, *bchpoly* can also return the factors *f* of the generator polynomial *p*, the cyclotomic coset for the Galois field over which the BCH code is calculated, the parity check matrix *par* and the error correction capability *t*. It should be noted that the parity check matrix is calculated with *cyclgen* and limitations in this function means that the parity check matrix is only available for codeword length upto 63. For codeword length longer than this *par* returns an empty matrix

With a string argument *probe* defined, the action of *bchpoly* is to calculate the error correcting capability of the BCH code defined by *n*, *k* and *prim* and return it in *p*. This is similar to a call to *bchpoly* with zero or one argument, except that only a single code is checked. Any string value for *probe* will force this action

In general the codeword length *n* can be expressed as `2^m-1`, where *m* is an integer. However, if [*n*,*k*] is a valid BCH code, then a shortened BCH code of the form [*n*-*x*,*k*-*x*] can be created with the same generator polynomial

See also: cyclpoly,encode,decode,cosets

9.2.10 bi2de

d = **bi2de** (*b*) Function File
d = **bi2de** (*b*,*p*) Function File
d = **bi2de** (*b*,*p*,*f*) Function File

Convert bit matrix to a vector of integers

Each row of the matrix *b* is treated as a single integer represented in binary form. The elements of *b*, must therefore be '0' or '1'

If *p* is defined then it is treated as the base of the decomposition and the elements of *b* must then lie between '0' and 'p-1'

The variable *f* defines whether the first or last element of *b* is considered to be the most-significant. Valid values of *f* are 'right-msb' or 'left-msb'. By default *f* is 'right-msb'

See also: de2bi

9.2.11 biterr

[*num*, *rate*] = **biterr** (*a*,*b*) Function File
[*num*, *rate*] = **biterr** (...,*k*) Function File
[*num*, *rate*] = **biterr** (...,*flag*) Function File
[*num*, *rate ind*] = **biterr** (...) Function File

Compares two matrices and returns the number of bit errors and the bit error rate. The binary representations of the variables *a* and *b* are treated and *a* and *b* can be either:

Both matrices

In this case both matrices must be the same size and then by default the the return values *num* and *rate* are the overall number of bit errors and the overall bit error rate

One column vector
: In this case the column vector is used for bit error comparision column-wise with the matrix. The returned values *num* and *rate* are then row vectors containing the num of bit errors and the bit error rate for each of the column-wise comparisons. The number of rows in the matrix must be the same as the length of the column vector

One row vector
: In this case the row vector is used for bit error comparision row-wise with the matrix. The returned values *num* and *rate* are then column vectors containing the num of bit errors and the bit error rate for each of the row-wise comparisons. The number of columns in the matrix must be the same as the length of the row vector

This behaviour can be overridden with the variable *flag*. *flag* can take the value 'column-wise', 'row-wise' or 'overall'. A column-wise comparision is not possible with a row vector and visa-versa

By default the number of bits in each symbol is assumed to be give by the number required to represent the maximum value of *a* and *b* The number of bits to represent a symbol can be overridden by the variable *k*

9.2.12 bsc

y = **bsc** (*data*, *p*) Function File

Send *data* into a binary symetric channel with probability *p* of error one each symbol

9.2.13 comms

comms (`'help'`) Function File
comms (`'info'`) Function File
comms (`'info'`, *mod*) Function File
comms (`'test'`) Function File
comms (`'test'`, *mod*) Function File

Manual and test code for the Octave Communications toolbox. There are 5 possible ways to call this function

`comms ('help')`
: Display this help message. Called with no arguments, this function also displays this help message

`comms ('info')`
: Open the Commumications toolbox manual

`comms ('info',` *mod*`)`
: Open the Commumications toolbox manual at the section specified by *mod*

`comms ('test')`
: Run all of the test code for the Communications toolbox

`comms ('test', mod)`
Run only the test code for the Communications toolbox in the module *mod*

Valid values for the varibale *mod* are

'all' All of the toolbox

'random' The random signal generation and analysis package

'source' The source coding functions of the package

'block' The block coding functions

'convol' The convolution coding package

'modulation'
The modulation package

'special' The special filter functions

'galois' The Galois fields package

Please note that this function file should be used as an example of the use of this toolbox

9.2.14 compand

y = **compand** (*x*, *mu*, *V*, `'mu/compressor'`) Function File
y = **compand** (*x*, *mu*, *V*, `'mu/expander'`) Function File
y = **compand** (*x*, *mu*, *V*, `'A/compressor'`) Function File
y = **compand** (*x*, *mu*, *V*, `'A/expander'`) Function File

Compresses and expanding the dynamic range of a signal using a mu-law or or A-law algorithm

The mu-law compressor/expander for reducing the dynamic range, is used if the fourth argument of *compand* starts with 'mu/'. Whereas the A-law compressor/expander is used if *compand* starts with 'A/' The mu-law algorithm uses the formulation

$$y = \frac{V log(1 + \mu/V x)}{log(1 + \mu)} sgn(x)$$

while the A-law algorithm used the formulation

$$y = \left| \begin{cases} A/(1 + logA)x, & 0 <= x <= V/A \\ \frac{V log(1 + log(A/V x))}{1 + logA}, & V/A < x <= V \end{cases} \right|$$

Neither converts from or to audio file ulaw format. Use mu2lin or lin2mu instead

See also: m2ulin, lin2mu

9.2.15 cosets

cosets (*m*, *prim*) Function File

Finds the elements of GF(2^*m*) with primitive polynomial *prim*, that share the same minimum polynomial. Returns a cell array of the paratitioning of GF(2^*m*)

9.2.16 cyclgen

h = **cyclgen** (*n*,*p*) Loadable Function
h = **cyclgen** (*n*,*p*,*typ*) Loadable Function
[*h*, *g*] = **cyclgen** (...) Loadable Function
[*h*, *g*, *k*] = **cyclgen** (...) Loadable Function

Produce the parity check and generator matrix of a cyclic code. The parity check matrix is returned as a *m* by *n* matrix, representing the [*n*,*k*] cyclic code. *m* is the order of the generator polynomial *p* and the message length *k* is given by *n* - *m*.

The generator polynomial can either be a vector of ones and zeros, and length *m* representing,

$$p_0 + p_1 x + p_2 x^2 + \cdots + p_m x^{m-1}$$

The terms of the polynomial are stored least-significant term first. Alternatively, *p* can be an integer representation of the same polynomial.

The form of the parity check matrix is determined by *typ*. If *typ* is 'system', a systematic parity check matrix is produced. If *typ* is 'nosys' and non-systematic parity check matrix is produced.

If requested *cyclgen* also returns the *k* by *n* generator matrix *g*.

See also: hammgen,gen2par,cyclpoly

9.2.17 cyclpoly

y = **cyclpoly** (*n*,*k*) Loadable Function
y = **cyclpoly** (*n*,*k*,*opt*) Loadable Function
y = **cyclpoly** (*n*,*k*,*opt*,*rep*) Loadable Function

This function returns the cyclic generator polynomials of the code [*n*,*k*]. By default the the polynomial with the smallest weight is returned. However this behavior can be overridden with the *opt* flag. Valid values of *opt* are:

'all' Returns all of the polynomials of the code [*n*,*k*]

'min' Returns the polynomial of minimum weight of the code [*n*,*k*]

'max' Returns the polynomial of the maximum weight of the code [*n*,*k*]

l Returns the polynomials having exactly the weight *l*

The polynomials are returns as row-vectors in the variable *y*. Each row of *y* represents a polynomial with the least-significant term first. The polynomials can be returned with an integer representation if *rep* is 'integer'. The default behaviour is given if *rep* is 'polynomial'.

See also: gf,isprimitive

9.2.18 de2bi

b = **de2bi** (*d*) Function File
b = **de2bi** (*d*,*n*) Function File
b = **de2bi** (*d*,*n*,*p*) Function File
b = **de2bi** (*d*,*n*,*p*,*f*) Function File

Convert a non-negative integer to bit vector

The variable *d* must be a vector of non-negative integers. *de2bi* then returns a matrix where each row represents the binary representation of elements of *d*. If *n* is defined then the returned matrix will have *n* columns. This number of columns can be either larger than the minimum needed and zeros will be added to the msb of the binary representation or smaller than the minimum in which case the least-significant part of the element is returned

If *p* is defined then it is used as the base for the decomposition of the returned values. That is the elements of the returned value are between '0' and 'p-1'

The variable *f* defines whether the first or last element of *b* is considered to be the most-significant. Valid values of *f* are 'right-msb' or 'left-msb'. By default *f* is 'right-msb'

See also: bi2de

9.2.19 decode

msg = **decode** (*code*,*n*,*k*) Function File
msg = **decode** (*code*,*n*,*k*,*typ*) Function File
msg = **decode** (*code*,*n*,*k*,*typ*,*opt1*) Function File
msg = **decode** (*code*,*n*,*k*,*typ*,*opt1*,*opt2*) Function File
[*msg*, *err*] = **decode** (...) Function File
[*msg*, *err*, *ccode*] = **decode** (...) Function File
[*msg*, *err*, *ccode*, *cerr*] = **decode** (...) Function File

Top level block decoder. This function makes use of the lower level functions such as *cyclpoly*, *cyclgen*, *hammgen*, and *bchenco*. The coded message to decode is pass in *code*, the codeword length is *n* and the message length is *k*. This function is used to decode messages using either:

A [n,k] linear block code defined by a generator matrix

A [n,k] cyclic code defined by a generator polynomial

A [n,k] Hamming code defined by a primitive polynomial

A [n,k] BCH code code defined by a generator polynomial

The type of coding to use is defined by the variable *typ*. This variable is a string taking one of the values

`'linear'` or `'linear/binary'`
: A linear block code is assumed with the message *msg* being in a binary format. In this case the argument *opt1* is the generator matrix, and is required. Additionally, *opt2* containing the syndrome lookup table (see *syndtable*) can also be passed

`'cyclic' or 'cyclic/binary'`
: A cyclic code is assumed with the message *msg* being in a binary format. The generator polynomial to use can be defined in *opt1* The default generator polynomial to use will be *cyclpoly(n,k)*. Additionally, *opt2* containing the syndrome lookup table (see *syndtable*) can also be passed

`'hamming' or 'hamming/binary'`
: A Hamming code is assumed with the message *msg* being in a binary format. In this case *n* must be of an integer of the form `2^m-1`, where *m* is an integer. In addition *k* must be *n-m*. The primitive polynomial to use can be defined in *opt1*. The default primitive polynomial to use is the same as defined by *hammgen*. The variable *opt2* should not be defined

`'bch' or 'bch/binary'`
: A BCH code is assumed with the message *msg* being in a binary format. The primitive polynomial to use can be defined in *opt2* The error correction capability of the code can also be defined in *opt1*. Use the empty matrix [] to let the error correction capability take the default value

In addition the argument 'binary' above can be replaced with 'decimal', in which case the message is assumed to be a decimal vector, with each value representing a symbol to be coded. The binary format can be in two forms

`An x-by-n matrix`
: Each row of this matrix represents a symbol to be decoded

`A vector with length divisible by n`
: The coded symbols are created from groups of *n* elements of this vector

The decoded message is return in *msg*. The number of errors encountered is returned in *err*. If the coded message format is 'decimal' or a 'binary' matrix, then *err* is a column vector having a length equal to the number of decoded symbols. If *code* is a 'binary' vector, then *err* is the same length as *msg* and indicated the number of errors in each symbol. If the value *err* is positive it indicates the number of errors corrected in the corresponding symbol. A negative value indicates an uncorrectable error. The corrected code is returned in *ccode* in a similar format to the coded message *msg*. The variable *cerr* contains similar data to *err* for *ccode*

It should be noted that all internal calculations are performed in the binary format. Therefore for large values of *n*, it is preferable to use the binary format to pass the messages to avoid possible rounding errors. Additionally, if repeated calls to *decode* will be performed, it is often faster to create a generator matrix externally with the functions *hammgen* or *cyclgen*, rather than let *decode* recalculate this matrix at each iteration. In this case *typ* should be 'linear'. The exception to this case is BCH codes, where the required syndrome table is too large. The BCH decoder, decodes directly from the polynomial never explicitly forming the syndrome table

See also: encode,cyclgen,cyclpoly,hammgen,bchdeco,bchpoly,syndtable

9.2.20 deintrlv

deintrlvd = **deintrlv** (*data*, *elements*) Function File

Restore elements of *data* according to *elements* See also: intrlv

9.2.21 demodmap

z = **demodmap** (*y*,*fd*,*fs*,'ask',*m*) Function File
z = **demodmap** (*y*,*fd*,*fs*,'fsk',*m*,*tone*) Function File
z = **demodmap** (*y*,*fd*,*fs*,'msk') Function File
z = **demodmap** (*y*,*fd*,*fs*,'psk',*m*) Function File
z = **demodmap** (*y*,*fd*,*fs*,'qask',*m*) Function File
z = **demodmap** (*y*,*fd*,*fs*,'qask/cir',*nsig*,*amp*,*phs*) Function File
z = **demodmap** (*y*,*fd*,*fs*,'qask/arb',*inphase*,*quadr*) Function File
z = **demodmap** (*y*,*fd*,*fs*,'qask/arb',*map*) Function File
z = **demodmap** (*y*,[*fd*, *off*],...) Function File

Demapping of an analog signal to a digital signal. The function *demodmap* must have at least three input arguments and one output argument. Argument *y* is a complex variable representing the analog signal to be demapped. The variables *fd* and *fs* are the sampling rate of the of digital signal and the sampling rate of the analog signal respectively. It is required that *fs*/*fd* is an integer

The available mapping of the digital signal are

'ask' Amplitude shift keying

'fsk' Frequency shift keying

'msk' Minimum shift keying

'psk' Phase shift keying

'qask'
'qsk'
'qam' Quadraure amplitude shift keying

In addition the 'qask', 'qsk' and 'qam' method can be modified with the flags '/cir' or '/arb'. That is 'qask/cir' and 'qask/arb', etc are valid methods and give circular- and arbitrary-qask mappings respectively. Also the method 'fsk' and 'msk' can be modified with the flag '/max', in which case *y* is assumed to be a matrix with *m* columns, representing the symbol correlations

The variable *m* is the order of the modulation to use. By default this is 2, and in general should be specified

For 'qask/cir', the additional arguments are the same as for *apkconst*, and you are referred to *apkconst* for the definitions of the additional variables

For 'qask/arb', the additional arguments *inphase* and *quadr* give the in-phase and quadrature components of the mapping, in a similar mapping to the outputs of *qaskenco* with one argument. Similar *map* represents the in-phase and quadrature components of the mapping as the real and imaginary parts of the variable *map*

See also: modmap,ddemodce,ademodce,apkconst,qaskenco

9.2.22 egolaydec

egolaydec (*R*) Function File

Given *R*, the received Extended Golay code, this function tries to decode *R* using the Extended Golay code parity check matrix Extended Golay code (24,12) which can correct upto 3 errors

The received code *R*, needs to be of length Nx24, for encoding. We can decode several codes at once, if they are stacked as a matrix of 24columns, each code in a separate row

The generator G used in here is same as obtained from the function egolaygen

The function returns the error-corrected code word from the received word. If decoding failed, the second return value is 1, otherwise it is 0

Extended Golay code (24,12) which can correct upto 3 errors. Decoding algorithm follows from Lin & Costello

Ref: Lin & Costello, pg 128, Ch4, 'Error Control Coding', 2nd ed, Pearson

```
M=[rand(10,12)>0.5];
C1=egolayenc(M);
C1(:,1)=mod(C1(:,1)+1,2)
C2=egolaydec(C1)
```

See also: egolaygen,egolayenc

9.2.23 egolayenc

egolayenc (*M*) Function File

Given *M*, encode M using the Extended Golay code

The message *M*, needs to be of size Nx12, for encoding We can encode several messages, into codes at once, if they are stacked in the order suggested

The generator G used in here is same as obtained from the function egolaygen. Extended Golay code (24,12) which can correct upto 3 errors

```
M=(rand(10,12)>0.5);
C=egolayenc(M)
```

See also: egolaygen,egolaydec

9.2.24 egolaygen

egolaygen () Function File

Returns the Extended Golay code (24,12) generator matrix, which can correct upto 3 errors. The second argument is the partiy check matrix, for this code

See also: egolaydec,egolayenc

9.2.25 encode

code = **encode** (*msg*,*n*,*k*) Function File
code = **encode** (*msg*,*n*,*k*,*typ*) Function File
code = **encode** (*msg*,*n*,*k*,*typ*,*opt*) Function File
[*code*, *added*] = **encode** (...) Function File

Top level block encoder. This function makes use of the lower level functions such as *cyclpoly*, *cyclgen*, *hammgen*, and *bchenco*. The message to code is pass in *msg*, the codeword length is *n* and the message length is *k*. This function is used to encode messages using either:

A [n,k] linear block code defined by a generator matrix

A [n,k] cyclic code defined by a generator polynomial

A [n,k] Hamming code defined by a primitive polynomial

A [n,k] BCH code code defined by a generator polynomial

The type of coding to use is defined by the variable *typ*. This variable is a string taking one of the values

`'linear'` or `'linear/binary'`
: A linear block code is assumed with the coded message *code* being in a binary format. In this case the argument *opt* is the generator matrix, and is required

`'cyclic'` or `'cyclic/binary'`
: A cyclic code is assumed with the coded message *code* being in a binary format. The generator polynomial to use can be defined in *opt* The default generator polynomial to use will be *cyclpoly(n,k)*

`'hamming'` or `'hamming/binary'`
: A Hamming code is assumed with the coded message *code* being in a binary format. In this case *n* must be of an integer of the form `2^`*m*`-1`, where *m* is an integer. In addition *k* must be *n*-*m*. The primitive polynomial to use can be defined in *opt*. The default primitive polynomial to use is the same as defined by *hammgen*

`'bch'` or `'bch/binary'`
: A BCH code is assumed with the coded message *code* being in a binary format. The generator polynomial to use can be defined in *opt* The default generator polynomial to use will be *bchpoly(n,k)*

In addition the argument 'binary' above can be replaced with 'decimal', in which case the message is assumed to be a decimal vector, with each value representing a symbol to be coded. The binary format can be in two forms

`An` *x*`-by-`*k* `matrix`
: Each row of this matrix represents a symbol to be coded

`A vector`
: The symbols are created from groups of *k* elements of this vector If the vector length is not divisble by *k*, then zeros are added and the number of zeros added is returned in *added*

It should be noted that all internal calculations are performed in the binary format. Therefore for large values of *n*, it is preferable to use the binary format to pass the messages to avoid possible rounding errors. Additionally, if repeated calls to *encode* will be performed, it is often faster to create a generator matrix externally with the functions *hammgen* or *cyclgen*, rather than let *encode* recalculate this matrix at each iteration. In this case *typ* should be 'linear'. The exception to this case is BCH codes, whose encoder is implemented directly from the polynomial and is significantly faster

See also: decode,cyclgen,cyclpoly,hammgen,bchenco,bchpoly

9.2.26 eyediagram

eyediagram (*x*,*n*) Function File
eyediagram (*x*,*n*,*per*) Function File
eyediagram (*x*,*n*,*per*,*off*) Function File
eyediagram (*x*,*n*,*per*,*off*,*str*) Function File
eyediagram (*x*,*n*,*per*,*off*,*str*,*h*) Function File
h = **eyediagram** (*...*) Function File

Plot the eye-diagram of a signal. The signal *x* can be either in one of three forms

A real vector
: In this case the signal is assumed to be real and represented by the vector *x*. A single eye-diagram representing this signal is plotted

A complex vector
: In this case the in-phase and quadrature components of the signal are plotted seperately

A matrix with two columns
: In this case the first column represents the in-phase and the second the quadrature components of a complex signal

Each line of the eye-diagram has *n* elements and the period is assumed to be given by *per*. The time axis is then [-*per*/2 *per*/2] By default *per* is 1

By default the signal is assumed to start at -*per*/2. This can be overridden by the *off* variable, which gives the number of samples to delay the signal

The string *str* is a plot style string (example 'r+'), and by default is the default gnuplot line style

The figure handle to use can be defined by *h*. If *h* is not given, then the next available figure handle is used. The figure handle used in returned on *hout*

See also: scatterplot

9.2.27 fibodeco

fibodeco (*code*) Function File

Returns the decoded fibonacci value from the binary vectors *code* Universal codes like fibonacci codes Have a useful synchronization property, only for 255 maximum

value we have designed these routines. We assume user has partitioned the code into several unique segments based on the suffix property of unique strings "11" and we just decode the parts. Partitioning the stream is as simple as identifying the "11" pairs that occur, at the terminating ends. This system implements the standard binaary Fibonacci codes, which means that row vectors can only contain 0 or 1. Ref: `http://en.wikipedia.org/wiki/Fibonacci_coding`

```
fibodeco({[0 1 0 0 1 1]}) %decoded to 10
fibodeco({[1 1],[0 1 1],[0 0 1 1],[1 0 1 1]}) %[1:4]
```

See also: fiboenco

9.2.28 fiboenco

fiboenco (*num*) Function File

Returns the cell-array of encoded fibonacci value from the column vectors *num* Universal codes like fibonacci codes have a useful synchronization property, only for 255 maximum value we have designed these routines. We assume user has partitioned the code into several unique segments based on the suffix property of unique elements [1 1] and we just decode the parts. Partitioning the stream is as simple as identifying the [1 1] pairs that occur, at the terminating ends. This system implements the standard binaary Fibonacci codes, which means that row vectors can only contain 0 or 1. Ref: http://en.wikipedia.org/wiki/Fibonacci_coding Ugly O(k.N^2) encoder.Ref: Wikipedia article accessed March, 2006 `http://en.wikipedia.org/wiki/Fibonacci_coding`, UCI Data Compression Book, `http://www.ics.uci.edu/~dan/pubs/DC-Sec3.html`, (accessed October 2006)

```
fiboenco(10) #=  code is {[ 0 1 0 0 1 1]}
fiboenco(1:4) #= code is {[1 1],[0 1 1],[0 0 1 1],[1 0 1 1]}
```

See also: fibodeco

9.2.29 fibosplitstream

fibosplitstream (*code*) Function File

Returns the split data stream at the word boundaries Assuming the stream was originally encoded using `fiboenco` and this routine splits the stream at the points where '11' occur together & gives us the code-words which can later be decoded from the `fibodeco` This however doesnt mean that we intend to verify if all the codewords are correct, and infact the last symbol in th return list can or can-not be a valid codeword

A example use of `fibosplitstream` would be

```
fibodeco(fibosplitstream([fiboenco(randint(1,100,[0 255])){:}]))
fibodeco(fibosplitstream([fiboenco(1:10){:}]))
```

See also: fiboenco,fibodeco

9.2.30 fmdemod

`fmdemod` (*x*,*fc*,*fs*) Function File

Create the FM demodulation of the signal x with carrier frequency fs. Where x is sample at frequency fs See also: ammod,amdemod,fmmod

9.2.31 fmmod

`fmmod` (*x*,*fc*,*fs*) Function File

Create the FM modulation of the signal x with carrier frequency fs. Where x is sample at frequency fs See also: ammod,fmdemod,amdemod

9.2.32 gconv

gconv (*a*, *b*) Function File

Convolve two Galois vectors

`y = gconv (a, b)` returns a vector of length equal to `length (a) + length (b) - 1` If *a* and *b* are polynomial coefficient vectors, `gconv` returns the coefficients of the product polynomial

See also: gdeconv,conv,deconv

9.2.33 gconvmtx

gconvmtx (*a*, *n*) Function File

Create matrix to perform repeated convolutions with the same vector in a Galois Field. If *a* is a column vector and *x* is a column vector of length *n*, in a Galois Field then

`gconvmtx(a, n) * x`

gives the convolution of of *a* and *x* and is the same as `gconv(a, x)`. The difference is if many vectors are to be convolved with the same vector, then this technique is possibly faster

Similarly, if *a* is a row vector and *x* is a row vector of length *n*, then

`x * gconvmtx(a, n)`

is the same as `gconv(x, a)`

See also: gconv,convmtx,conv

9.2.34 gdeconv

gdeconv (*y*, *a*) Function File

Deconvolve two Galois vectors

`[b, r] = gdeconv (y, a)` solves for *b* and *r* such that `y = gconv (a, b) + r`

If *y* and *a* are polynomial coefficient vectors, *b* will contain the coefficients of the polynomial quotient and *r* will be a remander polynomial of lowest order

See also: gconv,deconv,conv

9.2.35 gdet

d = **gdet** (*a*) Loadable Function

Compute the determinant of the Galois array *a*.

See also: det

9.2.36 gdftmtx

d = **gdftmtx** (*a*) Function File

Form a matrix, that can be used to perform Fourier transforms in a Galois Field

Given that *a* is an element of the Galois Field GF(2^m), and that the minimum value for *k* for which *a* ^ *k* is equal to one is `2^m - 1`, then this function produces a *k*-by-*k* matrix representing the discrete Fourier transform over a Galois Field with respect to *a*. The Fourier transform of a column vector is then given by `gdftmtx(a) * x`

The inverse Fourier transform is given by `gdftmtx(1/a)`

See also: dftmtx

9.2.37 gdiag

gdiag (*v*, *k*) Loadable Function

Return a diagonal matrix with Galois vector *v* on diagonal *k*. The second argument is optional. If it is positive, the vector is placed on the *k*-th super-diagonal. If it is negative, it is placed on the -*k*-th sub-diagonal. The default value of *k* is 0, and the vector is placed on the main diagonal. For example,

```
gdiag (gf([1, 2, 3],2), 1)
ans =
GF(2^2) array. Primitive Polynomial = D^2+D+1 (decimal 7)

Array elements =

  0  1  0  0
  0  0  2  0
  0  0  0  3
  0  0  0  0
```

See also: diag

9.2.38 gen2par

par = **gen2par** (*gen*) Function File
gen = **gen2par** (*par*) Function File

Converts binary generator matrix *gen* to the parity chack matrix *par* and visa-versa. The input matrix must be in standard form That is a generator matrix must be k-by-n and in the form [eye(k) P] or [P eye(k)], and the parity matrix must be (n-k)-by-n and of the form [eye(n-k) P'] or [P' eye(n-k)]

See also: cyclgen,hammgen

9.2.39 genqamdemod

y = **genqamdemod** (*x*, *C*) Loadable Function

General quadrature amplitude demodulation. The complex envelope quadrature amplitude modulated signal *x* is demodulated using a constellation mapping specified by the 1D vector *C*.

9.2.40 genqammod

y = **genqammod** (*x*, *c*) Function File

Modulates an information sequence of intergers *x* in the range `[0 ... M-1]` onto a quadrature amplitude modulated signal *y*, where `M = length(c) - 1` and *c* is a 1D vector specifing the signal constellation mapping to be used. An example of combined 4PAM-4PSK is

```
d = randint(1,1e4,8);
c = [1+j -1+j -1-j 1-j 1+sqrt(3) j*(1+sqrt(3)) -1-sqrt(3) -j*(1+sqrt(3))];
y = genqammod(d,c);
z = awgn(y,20);
plot(z,'rx')
```

See also: genqamdemod

9.2.41 gexp

gexp (*x*) Loadable Function

Compute the anti-logarithm for each element of *x* for a Galois array.

See also: exp

9.2.42 gf

y = **gf** (*x*) Loadable Function
y = **gf** (*x*, *m*) Loadable Function
y = **gf** (*x*, *m*, *primpoly*) Loadable Function

Creates a Galois field array GF(2^*m*) from the matrix *x*. The Galois field has 2^*m* elements, where *m* must be between 1 and 16. The elements of *x* must be between 0 and 2^*m* - 1. If *m* is undefined it defaults to the value 1.

The primitive polynomial to use in the creation of Galois field can be specified with the *primpoly* variable. If this is undefined a default primitive polynomial is used. It should be noted that the primitive polynomial must be of the degree *m* and it must be irreducible.

The output of this function is recognized as a Galois field by Octave and other matrices will be converted to the same Galois field when used in an arithmetic operation with a Galois field.

See also: isprimitive,primpoly

9.2.43 gfft

gfft (*x*) Function File

If *x* is a column vector, finds the FFT over the primitive element of the Galois Field of *x*. If *x* is in the Galois Field GF(2^*m*), then *x* must have `2^m - 1` elements

See also: fft

9.2.44 gfilter

y = **gfilter** (*b*, *a*, *x*) Loadable Function
[*y*, *sf*] = **gfilter** (*b*, *a*, *x*, *si*) Loadable Function

Digital filtering of vectors in a Galois Field. Returns the solution to the following linear, time-invariant difference equation over a Galois Field:

$$\sum_{k=0}^{N} a_{k+1} y_{n-k} = \sum_{k=0}^{M} b_{k+1} x_{n-k}, \qquad 1 \le n \le P$$

where $a \in \Re^{N-1}$, $b \in \Re^{M-1}$, and $x \in \Re^{P}$. An equivalent form of this equation is:

$$y_n = -\sum_{k=1}^{N} c_{k+1} y_{n-k} + \sum_{k=0}^{M} d_{k+1} x_{n-k}, \qquad 1 \le n \le P$$

where $c = a/a_1$ and $d = b/a_1$.

If the fourth argument *si* is provided, it is taken as the initial state of the system and the final state is returned as *sf*. The state vector is a column vector whose length is equal to the length of the longest coefficient vector minus one. If *si* is not supplied, the initial state vector is set to all zeros.

See also: filter

9.2.45 gftable

gftable (*m*, *primpoly*) Function File

This function exists for compatiability with matlab. As the octave galois fields store a copy of the lookup tables for every field in use internally, there is no need to use this function

See also: gf

9.2.46 gfweight

w = **gfweight** (*gen*) Function File
w = **gfweight** (*gen*,`'gen'`) Function File
w = **gfweight** (*par*,`'par'`) Function File
w = **gfweight** (*p*,`n`) Function File

Calculate the minimum weight or distance of a linear block code. The code can be either defined by its generator or parity check matrix, or its generator polynomial. By default if the first argument is a matrix, it is assumed to be the generator matrix of the code. The type of the matrix can be defined by a flag 'gen' for the generator matrix or 'par' for the parity check matrix

If the first argument is a vector, it is assumed that it defines the generator polynomial of the code. In this case a second argument is required that defines the codeword length

See also: hammgen,cyclpoly,bchpoly

9.2.47 gifft

gifft (*x*) Function File

If *x* is a column vector, finds the IFFT over the primitive element of the Galois Field of *x*. If *x* is in the Galois Field GF(2^*m*), then *x* must have `2^`*m* `- 1` elements

See also: ifft

9.2.48 ginv

[*x*, *rcond*] = **ginv** (*a*) Loadable Function

Compute the inverse of the square matrix *a*. Return an estimate of the reciprocal condition number if requested, otherwise warn of an ill-conditioned matrix if the reciprocal condition number is small.

See also: inv

9.2.49 ginverse

ginverse (*a*) Loadable Function

See ginv.

9.2.50 gisequal

gisequal (*x1*, *x2*, ...) Function File

Return true if all of *x1*, *x2*, ... are equal See also: isequalwithequalnans

9.2.51 glog

glog (*x*) Loadable Function

Compute the natural logarithm for each element of *x* for a Galois array.

See also: log

9.2.52 glu

[*l*, *u*, *p*] = **glu** (*a*) Loadable Function

Compute the LU decomposition of *a* in a Galois Field. The result is returned in a permuted form, according to the optional return value *p*. For example, given the matrix `a = gf([1, 2; 3, 4],3)`,

```
[l, u, p] = glu (a)
```

returns

```
l =
GF(2^3) array. Primitive Polynomial = D^3+D+1 (decimal 11)

Array elements =

  1  0
  6  1

u =
GF(2^3) array. Primitive Polynomial = D^3+D+1 (decimal 11)

Array elements =

  3  4
  0  7

p =

  0  1
  1  0
```

Such that $p * a = l * u$. If the argument *p* is not included then the permutations are applied to *l* so that $a = l * u$. *l* is then a pseudo- lower triangular matrix. The matrix *a* can be rectangular.

See also: lu

9.2.53 golombdeco

golombdeco (*code, m*) Function File

Returns the Golomb decoded signal vector using *code* and *m* Compulsory m is need to be specified. A restrictions is that a signal set must strictly be non-negative. The value of code is a cell array of row-vectors which have the encoded golomb value for a single sample. The Golomb algorithm is, used to encode the 'code' and only that can be meaningfully decoded. *code* is assumed to have been of format generated by the function `golombenco`. Also the parameter *m* need to be a non-zero number, unless which it makes divide-by-zero errors This function works backward the Golomb algorithm see `golombenco` for more detials on that Reference: Solomon Golomb, Run length Encodings, 1966 IEEE Trans Info' Theory

An exmaple of the use of `golombdeco` is

```
golombdeco(golombenco(1:4,2),2)
```

See also: golombenco

9.2.54 golombenco

golombenco (*sig, m*) Function File

Returns the Golomb coded signal as cell array Also total length of output code in bits can be obtained This function uses a *m* need to be supplied for encoding signal vector into a golomb coded vector. A restrictions is that a signal set must strictly be non-negative. Also the parameter *m* need to be a non-zero number, unless which it makes divide-by-zero errors The Golomb algorithm [1], is used to encode the data into unary coded quotient part which is represented as a set of 1's separated from the K-part (binary) using a zero. This scheme doesnt need any kind of dictionaries, it is a parameterized prefix codes Implementation is close to O(N^2), but this implementation *may be* sluggish, though correct. Details of the scheme are, to encode the remainder(r of number N) using the floor(log2(m)) bits when rem is in range 0:(2^ceil(log2(m)) - N), and encode it as r+(2^ceil(log2(m)) - N), using total of 2^ceil(log2(m)) bits in other instance it doesnt belong to case 1. Quotient is coded simply just using the unary code. Also accroding to [2] Golomb codes are optimal for sequences using the bernoulli probability model: P(n)=p^n-1.q & p+q=1, and when M=[1/log2(p)], or P=2^(1/M)

Reference: 1. Solomon Golomb, Run length Encodings, 1966 IEEE Trans Info' Theory. 2. Khalid Sayood, Data Compression, 3rd Edition

An exmaple of the use of `golombenco` is

```
golombenco(1:4,2) #
golombenco(1:10,2) #
```

See also: golombdeco

9.2.55 gprod

gprod (*x*, *dim*) Loadable Function

Product of elements along dimension *dim* of Galois array. If *dim* is omitted, it defaults to 1 (column-wise products).

See also: prod

9.2.56 grank

d = **grank** (*a*) Loadable Function

Compute the rank of the Galois array *a* by counting the independent rows and columns.

See also: rank

9.2.57 greshape

greshape (*a*, *m*, *n*) Loadable Function

Return a matrix with *m* rows and *n* columns whose elements are taken from the Galois array *a*. To decide how to order the elements, Octave pretends that the elements of a matrix are stored in column-major order (like Fortran arrays are stored).

For example,

```
greshape (gf([1, 2, 3, 4],3), 2, 2)
ans =
GF(2^3) array. Primitive Polynomial = D^3+D+1 (decimal 11)

Array elements =

  1  3
  2  4
```

The `greshape` function is equivalent to

```
retval = gf(zeros (m, n), a.m, a.prim_poly);
retval (:) = a;
```

but it is somewhat less cryptic to use `reshape` instead of the colon operator. Note that the total number of elements in the original matrix must match the total number of elements in the new matrix.

See also: reshape,':'

9.2.58 groots

groots (*v*) Function File

For a vector *v* with *N* components, return the roots of the polynomial over a Galois Field

$$v_1 z^{N-1} + \cdots + v_{N-1} z + v_N$$

The number of roots returned and their value will be determined by the order and primitive polynomial of the Galios Field

See also: roots

9.2.59 gsqrt

gsqrt (*x*) Loadable Function

Compute the square root of *x*, element by element, in a Galois Field.

See also: exp

9.2.60 gsum

gsum (*x*, *dim*) Loadable Function

Sum of elements along dimension *dim* of Galois array. If *dim* is omitted, it defaults to 1 (column-wise sum).

See also: sum

9.2.61 gsumsq

gsumsq (*x*, *dim*) Loadable Function

Sum of squares of elements along dimension *dim* of Galois array. If *dim* is omitted, it defaults to 1 (column-wise sum of squares).

This function is equivalent to computing

```
gsum (x .* conj (x), dim)
```

but it uses less memory.

See also: sumsq

9.2.62 hammgen

h = **hammgen** (*m*) Function File
h = **hammgen** (*m*,*p*) Function File
[*h*,*g*] = **hammgen** (...) Function File
[*h*,*g*,*n*,*k*] = **hammgen** (...) Function File

Produce the parity check and generator matrices of a Hamming code. The variable *m* defines the [*n*,*k*] Hamming code where *n* = 2 ^ *m* - 1 and *k* = *n* - *m* *m* must be between 3 and 16

The parity check matrix is generated relative to the primitive polynomial of GF(2^m). If *p* is specified the default primitive polynomial of GF(2^m) is overridden. *p* must be a valid primitive polynomial of the correct order for GF(2^m)

The parity check matrix is returned in the *m* by *n* matrix *h*, and if requested the generator matrix is returned in the *k* by *n* matrix *g*

See also: gen2par

9.2.63 huffmandeco

sig = **huffmandeco** (*hcode*, *dict*) Function File

Returns the original signal that was Huffman encoded signal using `huffmanenco`. This function uses a dict built from the `huffmandict` and uses it to decode a signal list into a huffman list. A restriction is that *hcode* is expected to be a binary code The returned signal set that strictly belongs in the range `[1,N]` with `N = length(dict)`. Also *dict* can only be from the `huffmandict` routine. Whenever decoding fails, those signal values a re indicated by `-1`, and we successively try to restart decoding from the next bit that hasn't failed in decoding, ad-infinitum. An exmaple of the use of `huffmandeco` is

```
hd = huffmandict(1:4,[0.5 0.25 0.15 0.10])
hcode = huffmanenco(1:4,hd) #  [ 1 0 1 0 0 0 0 0 1 ]
huffmandeco(hcode,h d) # [1 2 3 4]
```

See also: huffmandict, huffmanenco

9.2.64 huffmandict

huffmandict (*symb*, *prob*) Function File
huffmandict (*symb*, *prob*, *toggle*) Function File
huffmandict (*symb*, *prob*, *toggle*, *minvar*) Function File

Builds a Huffman code, given a probability list. The Huffman codes per symbol are output as a list of strings-per-source symbol. A zero probability symbol is NOT assigned any codeword as this symbol doesn't occur in practice anyway

toggle is an optional argument with values 1 or 0, that starts building a code based on 1's or 0's, defaulting to 0. Also *minvar* is a boolean value that is useful in choosing if you want to optimize buffer for transmission in the applications of Huffman coding, however it doesn't affect the type or average codeword length of the generated code. An example of the use of `huffmandict` is

```
huffmandict(symbols, [0.5 0.25 0.15 0.1]) => CW(0,10,111,110)
huffmandict(symbols, 0.25*ones(1,4)) => CW(11,10,01,00)

prob=[0.5 0 0.25 0.15 0.1]
dict=huffmandict(1:5,[0.5 0 0.25 0.15 0.1],1)
entropy(prob)
laverage(dict,prob)

x =   [0.20000   0.40000   0.20000   0.10000   0.10000];
illustrates the minimum variance thing
huffmandict(1,x,0,true) #min variance tree
huffmandict(1,x)     #normal huffman tree
```

Reference: Dr.Rao's course EE5351 Digital Video Coding, at UT-Arlington

See also: huffmandeco, huffmanenco

9.2.65 huffmanenco

huffmanenco (*sig*, *dict*) Function File

Returns the Huffman encoded signal using *dict*. This function uses a *dict* built from the `huffmandict` and uses it to encode a signal list into a huffman list. A restrictions is that a signal set must strictly belong in the range `[1,N]` with `N = length(dict)` Also *dict* can only be from the `huffmandict` routine An exmaple of the use of `huffmanenco` is

```
hd = huffmandict(1:4,[0.5 0.25 0.15 0.10])
huffmanenco(1:4,hd) #  [ 1 0 1 0 0 0 0 0 1 ]
```

See also: huffmandict, huffmandeco

9.2.66 intrlv

intrlvd = **intrlv** (*data*, *elements*) Function File

Interleaved elements of *data* according to *elements* See also: deintrlv

9.2.67 isgalois

isgalois (*expr*) Loadable Function

Return 1 if the value of the expression *expr* is a Galois Field.

9.2.68 isprimitive

y = **isprimitive** (*a*) Loadable Function

Returns 1 is the polynomial represented by *a* is a primitive polynomial of GF(2). Otherwise it returns zero.

See also: gf,primpoly

9.2.69 lloyds

[*table*, *codes*] = **lloyds** (*sig*,*init_codes*) Function File
[*table*, *codes*] = **lloyds** (*sig*,*len*) Function File
[*table*, *codes*] = **lloyds** (*sig*,...,*tol*) Function File
[*table*, *codes*] = **lloyds** (*sig*,...,*tol*,*type*) Function File
[*table*, *codes*, *dist*] = **lloyds** (...) Function File
[*table*, *codes*, *dist*, *reldist*] = **lloyds** (...) Function File

Optimize the quantization table and codes to reduce distortion. This is based on the article by Lloyd

S. Lloyd *Least squared quantization in PCM*, IEEE Trans Inform Thoery, Mar 1982, no 2, p129-137

which describes an iterative technique to reduce the quantization error by making the intervals of the table such that each interval has the same area under the PDF of the training signal *sig*. The initial codes to try can either be given in the vector *init_codes* or as scalar *len*. In the case of a scalar the initial codes will be an equi-spaced vector of length *len* between the minimum and maximum value of the training signal

The stopping criteria of the iterative algorithm is given by

```
abs(dist(n) - dist(n-1)) < max(tol, abs(eps*max(sig))
```

By default *tol* is 1.e-7. The final input argument determines how the updated table is created. By default the centroid of the values of the training signal that fall within the interval described by *codes* are used to update *table*. If *type* is any other string than "centroid", this behaviour is overriden and *table* is updated as follows

```
table = (code(2:length(code)) + code(1:length(code-1))) / 2
```

The optimized values are returned as *table* and *code*. In addition the distortion of the the optimized codes representing the training signal is returned as *dist*. The relative distortion in the final iteration is also returned as *reldist*

See also: quantiz

9.2.70 lz77deco

m = **lz77deco** (*c*, *alph*, *la*, *n*) Function File

Lempel-Ziv 77 source algorithm decoding implementation. Where

m message decoded (1xN)

c encoded message (Mx3)

alph size of alphabet

la lookahead buffer size

n sliding window buffer size

See also: lz77enco

9.2.71 lz77enco

c = **lz77enco** (*m*, *alph*, *la*, *n*) Function File

Lempel-Ziv 77 source algorithm implementation. Where

c encoded message (Mx3)

alph size of alphabet

la lookahead buffer size

n sliding window buffer size

See also: lz77deco

9.2.72 marcumq

[*Q*] = **marcumq** (*a*, *b*, *m*) Function File

Compute the Marcum Q function

9.2.73 matdeintrlv

intrlvd = **matdeintrlv** (*data*, *nrows*, *ncols*) Function File

Restore elements of *data* with a tempory matrix of size *nrows*-by-*ncols* See also: matintrlv

9.2.74 matintrlv

intrlvd = **matintrlv** (*data*, *nrows*, *ncols*) Function File

Interleaved elements of *data* with a tempory matrix of size *nrows*-by-*ncols* See also: matdeintrlv

9.2.75 minpol

minpol (*v*) Function File

Finds the minimum polynomial for elements of a Galois Field. For a vector *v* with *N* components, representing *N* values in a Galois Field GF(2^*m*), return the minimum polynomial in GF(2) representing thos values

9.2.76 modmap

modmap (*method*,*...*) Function File
y = **modmap** (*x*,*fd*,*fs*,'ask',*m*) Function File
y = **modmap** (*x*,*fd*,*fs*,'fsk',*m*,*tone*) Function File
y = **modmap** (*x*,*fd*,*fs*,'msk') Function File
y = **modmap** (*x*,*fd*,*fs*,'psk',*m*) Function File
y = **modmap** (*x*,*fd*,*fs*,'qask',*m*) Function File
y = **modmap** (*x*,*fd*,*fs*,'qask/cir',*nsig*,*amp*,*phs*) Function File
y = **modmap** (*x*,*fd*,*fs*,'qask/arb',*inphase*,*quadr*) Function File
y = **modmap** (*x*,*fd*,*fs*,'qask/arb',*map*) Function File

Mapping of a digital signal to an analog signal. With no output arguments *modmap* plots the constellation of the mapping. In this case the first argument must be the string *method* defining one of 'ask', 'fsk', 'msk', 'qask', 'qask/cir' or 'qask/arb'. The arguments following the string *method* are generally the same as those after the corresponding string in the fucntion call without output arguments The exception is `modmap('msk',Fd)`

With an output argument, *y* is the complex mapped analog signal. In this case the arguments *x*, *fd* and *fs* are required. The variable *x* is the digital signal to be mapped, *fd* is the sampling rate of the of digital signal and the *fs* is the sampling rate of the analog signal. It is required that *fs/fd* is an integer

The available mapping of the digital signal are

'ask' Amplitude shift keying

'fsk' Frequency shift keying

'msk' Minimum shift keying

'psk' Phase shift keying

'qask'
'qsk'
'qam' Quadraure amplitude shift keying

In addition the 'qask', 'qsk' and 'qam' method can be modified with the flags '/cir' or '/arb'. That is 'qask/cir' and 'qask/arb', etc are valid methods and give circular- and arbitrary-qask mappings respectively

The additional argument *m* is the order of the modulation to use *m* must be larger than the largest element of *x*. The variable *tone* is the FSK tone to use in the modulation

For 'qask/cir', the additional arguments are the same as for *apkconst*, and you are referred to *apkconst* for the definitions of the additional variables

For 'qask/arb', the additional arguments *inphase* and *quadr* give the in-phase and quadrature components of the mapping, in a similar mapping to the outputs of *qaskenco* with one argument. Similar *map* represents the in-phase and quadrature components of the mapping as the real and imaginary parts of the variable *map*

See also: demodmap,dmodce,amodce,apkconst,qaskenco

9.2.77 oct2dec

d = `oct2dec`(*c*) Function File

Convert octal to decimal values See also: base2dec,bin2dec,dec2bi

9.2.78 pamdemod

y = **pamdemod** (*x*, *m*) Function File
y = **pamdemod** (*x*, *m*, *phi*) Function File
y = **pamdemod** (*x*, *m*, *phi*, *type*) Function File

Demodulates a pulse amplitude modulated signal *x* into an information sequence of integers in the range `[0 ... M-1]` *phi* controls the initial phase and *type* controls the constellation mapping. If *type* is set to 'Bin' will result in binary encoding, in contrast, if set to 'Gray' will give Gray encoding An example of Gray-encoded 8-PAM is

```
d = randint(1,1e4,8);
y = pammod(d,8,0,'Gray');
z = awgn(y,20);
d_est = pamdemod(z,8,0,'Gray');
plot(z,'rx')
biterr(d,d_est)
```

See also: pammod

9.2.79 pammod

y = **pammod** (*x*, *m*) Function File
y = **pammod** (*x*, *m*, *phi*) Function File
y = **pammod** (*x*, *m*, *phi*, *type*) Function File

Modulates an information sequence of integers *x* in the range `[0 ... M-1]` onto a pulse amplitude modulated signal *y* *phi* controls the initial phase and *type* controls the constellation mapping. If *type* is set to 'Bin' will result in binary encoding, in contrast, if set to 'Gray' will give Gray encoding An example of Gray-encoded 8-PAM is

```
d = randint(1,1e4,8);
y = pammod(d,8,0,'Gray');
z = awgn(y,20);
plot(z,'rx')
```

See also: pamdemod

9.2.80 primpoly

y = **primpoly** (*m*) Loadable Function
y = **primpoly** (*m*, *opt*) Loadable Function
y = **primpoly** (*m*, ... 'nodisplay') Loadable Function

Finds the primitive polynomials in GF(2^*m*).

The first form of this function returns the default primitive polynomial of GF(2^*m*). This is the minimum primitive polynomial of the field. The polynomial representation is printed and an integer representation of the polynomial is returned

The call `primpoly (m, opt)` returns one or more primitive polynomials. The output of the function is dependent of the value of *opt*. Valid values of *opt* are:

'all' Returns all of the primitive polynomials of GF(2^*m*)

'min' Returns the minimum primitive polynomial of GF(2^*m*)

'max' Returns the maximum primitive polynomial of GF(2^*m*)

k Returns the primitive polynomials having exactly k non-zero terms

The call `primpoly (m, ... \'nodisplay\')` disables the output of the polynomial forms of the primitives. The return value is not affected.

See also: gf,isprimitive

9.2.81 pskdemod

y = **pamdemod** (*x*, *m*) Function File
y = **pamdemod** (*x*, *m*, *phi*) Function File
y = **pamdemod** (*x*, *m*, *phi*, *type*) Function File

Demodulates a complex-baseband phase shift keying modulated signal into an information sequence of integers in the range `[0 ... M-1]`. *phi* controls the initial phase and *type* controls the constellation mapping. If *type* is set to 'Bin' will result in binary encoding, in contrast, if set to 'Gray' will give Gray encoding. An example of Gray-encoded 8-PSK is

```
d = randint(1,1e3,8);
y = pskmod(d,8,0,'Gray');
z = awgn(y,20);
d_est = pskdemod(z,8,0,'Gray');
plot(z,'rx')
biterr(d,d_est)
```

See also: pskmod

9.2.82 pskmod

y = **pskmod** (*x*, *m*) Function File
y = **pskmod** (*x*, *m*, *phi*) Function File
y = **pskmod** (*x*, *m*, *phi*, *type*) Function File

Modulates an information sequence of integers *x* in the range `[0 ... M-1]` onto a complex baseband phase shift keying modulated signal *y*. *phi* controls the initial

phase and *type* controls the constellation mapping. If *type* is set to 'Bin' will result in binary encoding, in contrast, if set to 'Gray' will give Gray encoding. An example of Gray-encoded QPSK is

```
d = randint(1,5e3,4);
y = pskmod(d,4,0,'Gray');
z = awgn(y,30);
plot(z,'rx')
```

See also: pskdemod

9.2.83 qamdemod

`qamdemod` (*x*,*m*) Function File

Create the QAM demodulation of x with a size of alphabet m See also: qammod,pskmod,pskdemod

9.2.84 qammod

`qammod` (*x*,*m*) Function File

Create the QAM modulation of x with a size of alphabet m See also: qamdemod,pskmod,pskdemod

9.2.85 qaskdeco

msg = **qaskdeco** (*c*, *m*) Function File

msg = **qaskdeco** (*inphase*, *quadr*, *m*) Function File

msg = **qaskdeco** (*...*, *mnmx*) Function File

Demaps an analog signal using a square QASK constellation. The input signal maybe either a complex variable *c*, or as two real variables *inphase* and *quadr* representing the in-phase and quadrature components of the signal

The argument *m* must be a positive integer power of 2. By deafult the same constellation as created in *qaskenco* is used by *qaskdeco* If is possible to change the values of the minimum and maximum of the in-phase and quadrature components of the constellation to account for linear changes in the signal values in the received signal. The variable *mnmx* is a 2-by-2 matrix of the following form

```
| min in-phase      , max in-phase     |
| min quadrature    , max quadrature   |
```

If `sqrt(m)` is an integer, then *qaskenco* uses a Gray mapping. Otherwise, an attempt is made to create a nearly square mapping with a minimum Hamming distance between adjacent constellation points

See also: qaskenco

9.2.86 qaskenco

qaskenco (*m*) Function File
qaskenco (*msg*, *m*) Function File
y = **qaskenco** (...) Function File
[*inphase*, *quadr*] = **qaskenco** (...) Function File

Map a digital signal using a square QASK constellation. The argument *m* must be a positive integer power of 2. With two input arguments the variable *msg* represents the message to be encoded. The values of *msg* must be between 0 and *m*-1. In all cases `qaskenco(M)` is equivalent to `qaskenco(1:m,m)`

Three types of outputs can be created depending on the number of output arguments. That is

No output arguments
: In this case *qaskenco* plots the constellation. Only the points in *msg* are plotted, which in the case of a single input argument is all constellation points

A single output argument
: The returned variable is a complex variable representing the in-phase and quadrature components of the mapped message *msg*. With, a single input argument this effectively gives the mapping from symbols to constellation points

Two output arguments
: This is the same as two ouput arguments, expect that the in-phase and quadrature components are returned explicitly. That is

```
octave> c = qaskenco(msg, m);
octave> [a, b] = qaskenco(msg, m);
octave> all(c == a + 1i*b)
ans = 1
```

If `sqrt(m)` is an integer, then *qaskenco* uses a Gray mapping. Otherwise, an attempt is made to create a nearly square mapping with a minimum Hamming distance between adjacent constellation points

See also: qaskdeco

9.2.87 qfunc

[*y*] = **qfunc** (*x*) Function File

Compute the Q function See also: erfc, erf

9.2.88 qfuncinv

[*y*] = **qfuncinv** (*x*) Function File

Compute the inverse Q function See also: erfc, erf

9.2.89 quantiz

qidx = **quantiz** (*x*, *table*) Function File
[*qidx*, *q*] = **quantiz** (*x*, *table*, *codes*) Function File
[*qidx*, *q*, *d*] = **quantiz** (*...*) Function File

Quantization of an arbitrary signal relative to a paritioning

`qidx = quantiz(x, table)`
: Determine position of x in strictly monotonic table. The first interval, using index 0, corresponds to x <= table(1) Subsequent intervals are table(i-1) < x <= table(i)

`[qidx, q] = quantiz(x, table, codes)`
: Associate each interval of the table with a code. Use codes(1) for x <= table(1) and codes(n+1) for table(n) < x <= table(n+1)

`[qidx, q, d] = quantiz(...)`
: Compute distortion as mean squared distance of x from the corresponding quantization values

9.2.90 randdeintrlv

intrlvd = **randdeintrlv** (*data*, *state*) Function File

Restore elements of *data* with a random permutation See also: randintrlv,intrlv,deintrlv

9.2.91 randerr

b = **randerr** (*n*) Function File
b = **randerr** (*n*,*m*) Function File
b = **randerr** (*n*,*m*,*err*) Function File
b = **randerr** (*n*,*m*,*err*,*seed*) Function File

Generate a matrix of random bit errors. The size of the matrix is *n* rows by *m* columns. By default *m* is equal to *n* Bit errors in the matrix are indicated by a 1

The variable *err* determines the number of errors per row. By default the return matrix *b* has exactly one bit error per row If *err* is a scalar, there each row of *b* has exactly this number of errors per row. If *err* is a vector then each row has a number of errors that is in this vector. Each number of errors has an equal probability. If *err* is a matrix with two rows, then the first row determines the number of errors and the second their probabilities

The variable *seed* allows the random number generator to be seeded with a fixed value. The initial seed will be restored when returning

9.2.92 randint

b = **randint** (*n*) Function File
b = **randint** (*n*,*m*) Function File
b = **randint** (*n*,*m*,*range*) Function File
b = **randint** (*n*,*m*,*range*,*seed*) Function File

Generate a matrix of random binary numbers. The size of the matrix is *n* rows by *m* columns. By default *m* is equal to *n*

The range in which the integers are generated will is determined by the variable *range*. If *range* is an integer, the value will lie in the range [0,*range*-1], or [*range*+1,0] if *range* is negative. If *range* contains two elements the intgers will lie within these two elements, inclusive. By default *range* is assumed to be [0:1]

The variable *seed* allows the random number generator to be seeded with a fixed value. The initial seed will be restored when returning

9.2.93 randintrlv

intrlvd = **randintrlv** (*data*, *state*) Function File

Interleaves elements of *data* with a random permutation See also: intrlv,deintrlv

9.2.94 randsrc

b = **randsrc** (*n*) Function File
b = **randsrc** (*n*,*m*) Function File
b = **randsrc** (*n*,*m*,*alphabet*) Function File
b = **randsrc** (*n*,*m*,*alphabet*,*seed*) Function File

Generate a matrix of random symbols. The size of the matrix is *n* rows by *m* columns. By default *m* is equal to *n*

The variable *alphabet* can be either a row vector or a matrix with two rows. When *alphabet* is a row vector the symbols returned in *b* are chosen with equal probability from *alphabet*. When *alphabet* has two rows, the second row determines the probabilty with which each of the symbols is chosen. The sum of the probabilities must equal 1. By default *alphabet* is [-1 1]

The variable *seed* allows the random number generator to be seeded with a fixed value. The initial seed will be restored when returning

9.2.95 reedmullerdec

reedmullerdec (*VV*,*G*,*R*,*M*) Function File

Decode the received code word *VV* using the RM-generator matrix *G*, of order *R*, *M*, returning the code-word C. We use the standard majority logic vote method due to Irving S. Reed. The received word has to be a matrix of column size equal to to code-word size (i.e $2\hat{}m$). Each row is treated as a separate received word

The second return value is the message M got from C

G is obtained from definition type construction of Reed Muller code, of order R, length 2^M. Use the function reedmullergen, for the generator matrix for the (R,M) order RM code

Faster code constructions (also easier) exist, but since finding permutation order of the basis vectors, is important, we stick with the standard definitions. To use decoder function reedmullerdec, you need to use this specific generator function

see: Lin & Costello, Ch.4, "Error Control Coding", 2nd Ed, Pearson

```
G=reedmullergen(2,4);
M=[rand(1,11)>0.5];
C=mod(M*G,2);
[dec_C,dec_M]=reedmullerdec(C,G,2,4)
```

See also: reedmullergen,reedmullerenc

9.2.96 reedmullerenc

reedmullerenc (*MSG*,*R*,*M*) Function File

Definition type construction of Reed Muller code, of order R, length 2^M. This function returns the generator matrix for the said order RM code

Encodes the given message word/block, of column size k, corresponding to the RM(R,M), and outputs a code matrix C, on each row with corresponding codeword The second return value is the G, which is generator matrix used for this code

```
MSG=[rand(10,11)>0.5];
[C,G]=reedmullerenc(MSG,2,4);
```

See also: reedmullerdec,reedmullergen

9.2.97 reedmullergen

reedmullergen (*R*,*M*) Function File

Definition type construction of Reed Muller code, of order R, length 2^M. This function returns the generator matrix for the said order RM code

RM(r,m) codes are characterized by codewords, `sum ( (m,0) + (m,1) + ... + (m,r)` Each of the codeword is got through spanning the space, using the finite set of m-basis codewords Each codeword is 2^M elements long see: Lin & Costello, "Error Control Coding", 2nd Ed

Faster code constructions (also easier) exist, but since finding permutation order of the basis vectors, is important, we stick with the standard definitions. To use decoder function reedmullerdec, you need to use this specific generator function

```
G=reedmullergen(2,4);
```

See also: reedmullerdec,reedmullerenc

9.2.98 ricedeco

ricedeco (*code*, *K*) Function File

Returns the Rice decoded signal vector using *code* and *K* Compulsory K is need to be specified A restrictions is that a signal set must strictly be non-negative The value of code is a cell array of row-vectors which have the encoded rice value for a single sample. The Rice algorithm is used to encode the 'code' and only that can be meaningfully decoded. *code* is assumed to have been of format generated by the function `riceenco`

Reference: Solomon Golomb, Run length Encodings, 1966 IEEE Trans Info' Theory

An exmaple of the use of `ricedeco` is

```
ricedec(riceenco(1:4,2),2)
```

See also: riceenco

9.2.99 riceenco

riceenco (*sig*, *K*) Function File

Returns the Rice encoded signal using *K* or optimal K Default optimal K is chosen between 0-7. Currently no other way to increase the range except to specify explicitly. Also returns *K* parameter used (in case it were to be chosen optimally) and *Ltot* the total length of output code in bits This function uses a *K* if supplied or by default chooses the optimal K for encoding signal vector into a rice coded vector A restrictions is that a signal set must strictly be non-negative The Rice algorithm is used to encode the data into unary coded quotient part which is represented as a set of 1's separated from the K-part (binary) using a zero. This scheme doesnt need any kind of dictionaries and its close to O(N), but this implementation *may be* sluggish, though correct

Reference: Solomon Golomb, Run length Encodings, 1966 IEEE Trans Info' Theory

An exmaple of the use of `riceenco` is

```
riceenco(1:4) #
riceenco(1:10,2) #
```

See also: ricedeco

9.2.100 rledeco

rledeco (*message*) Function File

Returns decoded run-length *message* The RLE encoded *message* has to be in the form of a row-vector. The message format (encoded RLE) is like repetition [factor, value]+

An example use of `rledeco` is

```
message=[1 5 2 4 3 1];
rledeco(message) #gives
ans = [    5   4   4   1   1   1]
```

See also: rledeco

9.2.101 rleenco

rleenco (*message*) Function File

Returns run-length encoded *message*. The rle form is built from *message*. The original *message* has to be in the form of a row-vector. The encoded *message* format (encoded RLE) is like [repetition factor]+, values

An example use of `rleenco` is

```
message=[    5   4   4   1   1   1]
rleenco(message) #gives
ans = [1 5 2 4 3 1];
```

See also: rleenco

9.2.102 rsdec

msg = **rsdec** (*code*,*n*,*k*) Loadable Function
msg = **rsdec** (*code*,*n*,*k*,*g*) Loadable Function
msg = **rsdec** (*code*,*n*,*k*,*fcr*,*prim*) Loadable Function
msg = **rsdec** (...,*parpos*) Loadable Function
[*msg*,*nerr*]= **rsdec** (...) Loadable Function
[*msg*,*nerr*,*ccode*]= **rsdec** (...) Loadable Function

Decodes the message contained in *code* using a [*n*,*k*] Reed-Solomon code. The variable *code* must be a Galois array with *n* columns and an arbitrary number of rows. Each row of *code* represents a single block to be decoded by the Reed-Solomon coder. The decoded message is returned in the variable *msg* containing *k* columns and the same number of rows as *code*.

If *n* does not equal `2^`*m*`-1`, where m is an integer, then a shorten Reed-Solomon decoding is used where zeros are added to the start of each row to obtain an allowable codeword length. The returned *msg* has these prepending zeros stripped.

By default the generator polynomial used in the Reed-Solomon coding is based on the properties of the Galois Field in which *msg* is given. This default generator polynomial can be overridden by a polynomial in *g*. Suitable generator polynomials can be constructed with *rsgenpoly*. *fcr* is an integer value, and it is taken to be the first consecutive root of the generator polynomial. The variable *prim* is then the primitive element used to construct the generator polynomial. By default *fcr* and *prim* are both 1. It is significantly faster to specify the generator polynomial in terms of *fcr* and *prim*, since *g* is converted to this form in any case.

By default the parity symbols are placed at the end of the coded message. The variable *parpos* controls this positioning and can take the values 'beginning' or 'end'. If the parity symbols are at the end, the message is treated with the most-significant symbol first, otherwise the message is treated with the least-significant symbol first.

See also: gf,rsenc,rsgenpoly

9.2.103 rsdecof

rsdecof (*in*, *out*) Function File
rsdecof (*in*, *out*, *t*) Function File

Decodes an ascii file using a Reed-Solomon coder. The input file is defined by *in* and the result is written to the output file *out* The type of coding to use is determined by whether the input file is 7- or 8-bit. If the input file is 7-bit, the default coding is [127,117] while the default coding for an 8-bit file is a [255, 235]. This allows for 5 or 10 error characters in 127 or 255 symbols to be corrected respectively. The number of errors that can be corrected can be overridden by the variable *t*

If the file is not an integer multiple of the message size (127 or 255) in length, then the file is padded with the EOT (ascii character 4) character before decoding

See also: rsencof

9.2.104 rsenc

code = **rsenc** (*msg*, *n*, *k*) Loadable Function
code = **rsenc** (*msg*, *n*, *k*, *g*) Loadable Function
code = **rsenc** (*msg*, *n*, *k*, *fcr*, *prim*) Loadable Function
code = **rsenc** (*...*, *parpos*) Loadable Function

Encodes the message *msg* using a [*n*,*k*] Reed-Solomon coding. The variable *msg* is a Galois array with *k* columns and an arbitrary number of rows. Each row of *msg* represents a single block to be coded by the Reed-Solomon coder. The coded message is returned in the Galois array *code* containing *n* columns and the same number of rows as *msg*.

The use of *rsenc* can be seen in the following short example.

```
m = 3; n = 2^m -1; k = 3;
msg = gf([1 2 3; 4 5 6], m);
code = rsenc(msg, n, k);
```

If *n* does not equal `2^`*m*`-1`, where m is an integer, then a shorten Reed-Solomon coding is used where zeros are added to the start of each row to obtain an allowable codeword length. The returned *code* has these prepending zeros stripped.

By default the generator polynomial used in the Reed-Solomon coding is based on the properties of the Galois Field in which *msg* is given. This default generator polynomial can be overridden by a polynomial in *g*. Suitable generator polynomials can be constructed with *rsgenpoly*. *fcr* is an integer value, and it is taken to be the first consecutive root of the generator polynomial. The variable *prim* is then the primitive element used to construct the generator polynomial, such that $g = (x - A^b)(x - A^{b+p}) \cdots (x - A^{b+2tp-1})$.

where b is equal to *fcr* `*` *prim*. By default *fcr* and *prim* are both 1.

By default the parity symbols are placed at the end of the coded message. The variable *parpos* controls this positioning and can take the values 'beginning' or 'end'.

See also: gf,rsdec,rsgenpoly

9.2.105 rsencof

rsencof (*in*, *out*) Function File
rsencof (*in*, *out*, *t*) Function File
rsencof (..., *pad*) Function File

Encodes an ascii file using a Reed-Solomon coder. The input file is defined by *in* and the result is written to the output file *out* The type of coding to use is determined by whether the input file is 7- or 8-bit. If the input file is 7-bit, the default coding is [127,117] while the default coding for an 8-bit file is a [255, 235]. This allows for 5 or 10 error characters in 127 or 255 symbols to be corrected respectively. The number of errors that can be corrected can be overridden by the variable *t*

If the file is not an integer multiple of the message size (127 or 255) in length, then the file is padded with the EOT (ascii character 4) characters before coding. Whether these characters are written to the output is defined by the *pad* variable. Valid values for *pad* are `"pad"` (the default) and `"nopad"`, which write or not the padding respectively

See also: rsdecof

9.2.106 rsgenpoly

g = **rsgenpoly** (*n*, *k*) Function File
g = **rsgenpoly** (*n*, *k*, *p*) Function File
g = **rsgenpoly** (*n*, *k*, *p*, *b*, *s*) Function File
g = **rsgenpoly** (*n*, *k*, *p*, *b*) Function File
[*g*, *t*] = **rsgenpoly** (...) Function File

Creates a generator polynomial for a Reed-Solomon coding with message length of *k* and codelength of *n*. *n* must be greater than *k* and their difference must be even. The generator polynomial is returned on *g* as a polynomial over the Galois Field GF(2^*m*) where *n* is equal to `2^m-1`. If *m* is not integer the next highest integer value is used and a generator for a shorten Reed-Solomon code is returned

The elements of *g* represent the coefficients of the polynomial in descending order. If the length of *g* is lg, then the generator polynomial is given by

$$g_0 x^{lg-1} + g_1 x^{lg-2} + \cdots + g_{lg-1} x + g_l g$$

If *p* is defined then it is used as the primitive polynomial of the the Galois Field GF(2^*m*). The default primitive polynomial will be used if *p* is equal to []

The variables *b* and *s* determine the form of the generator polynomial in the following manner

$$g = (x - A^{bs})(x - A^{(b+1)s}) \cdots (x - A^{(b+2t-1)s})$$

where *t* is `(n-k)/2`, and A is the primitive element of the Galois Field. Therefore *b* is the first consecutive root of the generator polynomial and *s* is the primitive element to generate the the polynomial roots

If requested the variable *t*, which gives the error correction capability of the the Reed-Solomon code

See also: gf,rsenc,rsdec

9.2.107 scatterplot

scatterplot (*x*) Function File
scatterplot (*x*,*n*) Function File
scatterplot (*x*,*n*,*off*) Function File
scatterplot (*x*,*n*,*off*,*str*) Function File
scatterplot (*x*,*n*,*off*,*str*,*h*) Function File
h = **scatterplot** (*...*) Function File

Display the scatter plot of a signal. The signal *x* can be either in one of three forms

A real vector
: In this case the signal is assumed to be real and represented by the vector *x*. The scatterplot is plotted along the x axis only

A complex vector
: In this case the in-phase and quadrature components of the signal are plotted seperately on the x and y axes respectively

A matrix with two columns
: In this case the first column represents the in-phase and the second the quadrature components of a complex signal and are plotted on the x and y axes respectively

Each point of the scatter plot is assumed to be seperated by *n* elements in the signal. The first element of the signal to plot is determined by *off*. By default *n* is 1 and *off* is 0

The string *str* is a plot style string (example 'r+'), and by default is the default gnuplot point style

The figure handle to use can be defined by *h*. If *h* is not given, then the next available figure handle is used. The figure handle used in returned on *hout*

See also: eyediagram

9.2.108 shannonfanodeco

shannonfanodeco (*hcode*,*dict*) Function File

Returns the original signal that was Shannonfano encoded. The signal was encoded using `shannonfanoenco`. This function uses a dict built from the `shannonfanodict` and uses it to decode a signal list into a shannonfano list. Restrictions include hcode is expected to be a binary code; returned signal set that strictly belongs in the `range [1,N]`, with `N=length(dict)`. Also dict can only be from the `shannonfanodict(...)` routine. Whenever decoding fails, those signal values are indicated by -1, and we successively try to restart decoding from the next bit that hasnt failed in decoding, ad-infinitum

An example use of `shannonfanodeco` is

```
hd=shannonfanodict(1:4,[0.5 0.25 0.15 0.10])
hcode=shannonfanoenco(1:4,hd) #  [ 1   0   1   0   0   0   0   0   1 ]
shannonfanodeco(hcode,hd) # [1 2 3 4]
```

See also: shannonfanoenco, shannonfanodict

9.2.109 shannonfanodict

shannonfanodict (*symbols*, *symbol_probabilites*) Function File

Returns the code dictionary for source using shanno fano algorithm Dictionary is built from *symbol_probabilities* using the shannon fano scheme. Output is a dictionary cell-array, which are codewords, and correspond to the order of input probability

```
CW=shannonfanodict(1:4,[0.5 0.25 0.15 0.1]);
assert(redundancy(CW,[0.5 0.25 0.15 0.1]),0.25841,0.001)
shannonfanodict(1:5,[0.35 0.17 0.17 0.16 0.15])
shannonfanodict(1:8,[8 7 6 5 5 4 3 2]./40)
```

See also: shannonfanoenc, shannonfanodec

9.2.110 shannonfanoenco

shannonfanoenco (*hcode*, *dict*) Function File

Returns the Shannon Fano encoded signal using *dict* This function uses a *dict* built from the `shannonfanodict` and uses it to encode a signal list into a shannon fano code Restrictions include a signal set that strictly belongs in the `range [1,N]` with `N=length(dict)`. Also dict can only be from the `shannonfanodict()` routine An example use of `shannonfanoenco` is

```
hd=shannonfanodict(1:4,[0.5 0.25 0.15 0.10])
shannonfanoenco(1:4,hd) #  [   0   1   0   1   1   0   1   1   1   0]
```

See also: shannonfanodeco, shannonfanodict

9.2.111 symerr

[*num*, *rate*] = **symerr** (*a*, *b*) Function File
[*num*, *rate*] = **symerr** (..., *flag*) Function File
[*num*, *rate ind*] = **symerr** (...) Function File

Compares two matrices and returns the number of symbol errors and the symbol error rate. The variables *a* and *b* can be either:

Both matrices
: In this case both matrices must be the same size and then by default the the return values *num* and *rate* are the overall number of symbol errors and the overall symbol error rate

One column vector
: In this case the column vector is used for symbol error comparision column-wise with the matrix. The returned values *num* and *rate* are then row vectors containing the num of symbol errors and the symbol error rate for each of the column-wise comparisons. The number of rows in the matrix must be the same as the length of the column vector

One row vector

In this case the row vector is used for symbol error comparision row-wise with the matrix. The returned values *num* and *rate* are then column vectors containing the num of symbol errors and the symbol error rate for each of the row-wise comparisons. The number of columns in the matrix must be the same as the length of the row vector

This behaviour can be overridden with the variable *flag*. *flag* can take the value 'column-wise', 'row-wise' or 'overall'. A column-wise comparision is not possible with a row vector and visa-versa

9.2.112 syndtable

t = **syndtable** (*h*) Loadable Function

Create the syndrome decoding table from the parity check matrix *h*. Each row of the returned matrix *t* represents the error vector in a recieved symbol for a certain syndrome. The row selected is determined by a conversion of the syndrome to an integer representation, and using this to reference each row of *t*.

See also: hammgen,cyclgen

9.2.113 systematize

systematize (*G*) Function File

Given *G*, extract P partiy check matrix. Assume row-operations in GF(2) *G* is of size KxN, when decomposed through row-operations into a *I* of size KxK identity matrix, and a parity check matrix *P* of size Kx(N-K)

Most arbitrary code with a given generator matrix *G*, can be converted into its systematic form using this function

This function returns 2 values, first is default being *Gx* the systematic version of the *G* matrix, and then the parity check matrix *P*

```
G=[1 1 1 1; 1 1 0 1; 1 0 0 1];
[Gx,P]=systematize(G);

Gx = [1 0 0 1; 0 1 0 0; 0 0 1 0];
P = [1 0 0];
```

See also: bchpoly,biterr

9.2.114 vec2mat

m = **vec2mat** (*v*, *c*) Function File
m = **vec2mat** (*v*, *c*, *d*) Function File
[*m*, *add*] = **vec2mat** (...) Function File

Converts the vector *v* into a *c* column matrix with row priority arrangement and with the final column padded with the value *d* to the correct length. By default *d* is 0. The amount of padding added to the matrix is returned in *add*

9.2.115 wgn

y = **wgn** (*m*,*n*,*p*) Function File
y = **wgn** (*m*,*n*,*p*,*imp*) Function File
y = **wgn** (*m*,*n*,*p*,*imp*,*seed*,) Function File
y = **wgn** (...,'*type*') Function File
y = **wgn** (...,'*output*') Function File

Returns a M-by-N matrix *y* of white Gaussian noise. *p* specifies the power of the output noise, which is assumed to be referenced to an impedance of 1 Ohm, unless *imp* explicitly defines the impedance

If *seed* is defined then the randn function is seeded with this value

The arguments *type* and *output* must follow the above numerial arguments, but can be specified in any order. *type* specifies the units of *p*, and can be 'dB', 'dBW', 'dBm' or 'linear'. 'dB' is in fact the same as 'dBW' and is keep as a misnomer of Matlab. The units of 'linear' are in Watts

The *output* variable should be either 'real' or 'complex'. If the output is complex then the power *p* is divided equally betwen the real and imaginary parts

See also: randn,awgn
